# Enhancing the toolbox to study IL-17A in cattle and sheep

**DOI:** 10.1186/s13567-017-0426-5

**Published:** 2017-04-08

**Authors:** Sean R. Wattegedera, Yolanda Corripio-Miyar, Yvonne Pang, David Frew, Tom N. McNeilly, Javier Palarea-Albaladejo, Colin J. McInnes, Jayne C. Hope, Elizabeth J. Glass, Gary Entrican

**Affiliations:** 1 Moredun Research Institute, International Research Centre, Pentlands Science Park, Bush Loan, Penicuik, Scotland EH26 0PZ UK; 2 The Roslin Institute and Royal (Dick) School of Veterinary Studies, The University of Edinburgh, Easter Bush, Midlothian, Scotland EH25 9RG UK; 3 Biomathematics and Statistics Scotland, JCMB, The King’s Buildings, Peter Guthrie Tait Road, Edinburgh, Scotland EH9 3FD UK

## Abstract

The development of methods to detect cytokine expression by T cell subsets in ruminants is fundamental to strategic development of new livestock vaccines for prevention of infectious diseases. It has been possible to detect T cell expression of IFN-γ, IL-4 and IL-10 in ruminants for many years but methods to detect expression of IL-17A are relatively limited. To address this gap in capability we have cloned bovine and ovine IL-17A cDNAs and expressed biologically-active recombinant proteins in Chinese Hamster Ovary (CHO) cells. We used the transfected CHO cells to screen commercially-available antibodies for their ability to detect IL-17A expression intracellularly and in culture supernates. We demonstrate that an ELISA for bovine IL-17A detects native ovine IL-17A. Moreover, the constituent polyclonal antibodies (pabs) in the ELISA were used to enumerate peripheral blood mononuclear cells (PBMC) expressing IL-17A from cattle and sheep by ELISpot. We identified two monoclonal antibodies (mabs) that detect recombinant intracellular IL-17A in CHO cells by flow cytometry. One of these mabs was used to detect native intracellular IL-17A expression in PBMC in conjunction with cell surface phenotyping mabs [CD4+ve, CD8+ve and Workshop Cluster 1 (WC-1)+ve gamma-delta (γδ)] we show that distinct T cell subsets in cattle (defined as CD4+ve, CD8+ve or WC-1+ve) and sheep (defined as CD4+ve or WC-1+ve) can express IL-17A following activation. These novel techniques provide a solid basis to investigate IL-17A expression and define specific CD4+ve T cell subset activation in ruminants.

**Electronic supplementary material:**

The online version of this article (doi:10.1186/s13567-017-0426-5) contains supplementary material, which is available to authorized users.

## Introduction

Interleukin(IL)-17 was first described in 1993 as a novel transcript in a T cell hybridoma clone and named cytotoxic T lymphocyte associated antigen 8 (CTLA-8) [1]. It was subsequently renamed IL-17A and it is one of the IL-17 family of six related homodimeric cytokines [IL-17A, -B, -C, -D, -E (also known as IL-25) and -F] that are involved in acute and chronic inflammatory responses in humans and murine models as reviewed by Gu et al. [2]. IL-17A is the “signature cytokine” secreted by the Th-17 CD4+ve T cell subset [3]. Activation of Th-17-type responses are important not only for host immune control of extracellular bacterial and fungal infections but are also associated with chronic inflammation and autoimmunity. Detailed knowledge of IL-17A biology in humans has led to the targeted development of immunotherapeutic monoclonal antibodies (mabs) to block IL-17A and the IL-17RA receptor for the treatment and control of psoriasis, multiple sclerosis and rheumatoid arthritis [4].

However, as for many immunological parameters, our knowledge of IL-17A production and its function in veterinary species is very limited compared to humans and biomedical rodent models [5] despite cloning of bovine IL-17A in 2006 [6]. In farmed ruminant species, there are published studies that measure mRNA encoding bovine IL-17 family members. These include IL-17A expression in purified protein derivative-stimulated peripheral blood mononuclear cells (PBMC) from cattle with macroscopic lung lesion pathology following experimental *Mycobacterium bovis* infection [7]; IL-17A and IL-17F in afferent lymph cells in response to liposomal vaccine preparations [8]; and IL-17A, IL-17C, IL-17E and IL-17F in the udder of lactating cows infected with *Escherichia coli* [9]. Measurement of IL-17 family members at the protein level in ruminant species has been limited by the paucity of species-specific reagents with the exception of one commercially-available ELISA kit to detect bovine IL-17A (Kingfisher Biotech). Using this ELISA, Flynn et al. [10] have shown the capacity of *Neospora caninum*-infected bovine macrophages to stimulate IL-17A production in naive CD4+ve T cells while Tassi et al. [11] have shown the presence of IL-17A in milk from lactating cattle infected by *Streptococcus uberis*.

The detection and biological function of other IL-17 family members in ruminants is less well characterised. Bougarn et al. [12] and Roussel et al. [9] reported the up-regulation of mRNA encoding the chemokines CCL2 and CCL20 in primary bovine mammary epithelial cells by recombinant bovine IL-17A and IL-17F, consistent with activities of the human and rodent orthologues. In 2011, Gossner et al. [13] sequenced two variant IL-17E (IL-25) sequences from ovine gastric lymph nodes. Most of the cattle, sheep and goat IL-17 family sequences have been published (Additional file 1) but only recombinant IL-17A is commercially available at present for all three species, with IL-17F also available for cattle. To improve our capability to study IL-17A biology in sheep and cattle we have screened a panel of commercial antibodies for ability to recognise recombinant bovine and ovine IL-17A expressed in mammalian cells and further evaluated their performance in a range of techniques for detection of the native cytokine in T cell subsets.

## Materials and methods

### Animals

Four Texel-cross ewes of approximately 3 years of age, six Greyface ewes 2–4 years of age and three male Holstein–Friesian cattle 8–12 months of age were maintained off pasture by the Bioservices Division at Moredun Research Institute (MRI) in helminth-free conditions. In addition, four female Holstein–Friesian cattle of approximately 2 years of age were maintained off pasture at Dryden Farm, The Roslin Institute (RI) at The University of Edinburgh. All animal procedures were approved by the Ethics Committees at MRI and RI and performed to Home Office Guidelines under Project Licences PPL 60/4394, PPL 60/3854, PPL 60/4380 and PPL 60/4391. Venous blood was collected into heparinised vacutainers (Becton–Dickinson, Oxford, UK) or into syringes containing sodium heparin (final concentration 20 U/mL, Sigma-Aldrich, Dorset, UK). PBMC were isolated from heparinised sheep and cattle blood by density centrifugation using established protocols [14] and used for molecular cloning, mitogen re-stimulation assays and ELISpot assays. For analysis of cytokine expression by specific T cell subsets, an additional red blood cell lysis step was applied to the PBMC following density centrifugation using a lysis buffer containing 10 mM KHCO_3_, 150 mM NH_4_Cl, 0.1 mM EDTA pH 8.0 for 5 min at room temperature (RT).

### Cloning, sequencing and bioinformatic analyses of IL-17A

We have observed that Concanavalin A (ConA) stimulates ruminant PBMC and found it very effective at upregulating mRNA expression of inflammatory (IFN-γ) and regulatory (IL-10) cytokines from 6 to 96 h [15] and considered this time-frame ideal for acquiring template cDNA for bovine and ovine IL-17A. PBMC from one male calf (male Holstein–Friesian) and one ewe (Texel-cross) were seeded into six-well flat bottom plates (Corning Costar, Scientific Laboratory Supplies Ltd, Coatbridge, UK) at a concentration of 1 × 10^6^ cells/mL in RPMI-1640 (Gibco, Life Technologies, Paisley, UK) supplemented with 0.1% 2-mercaptoethanol (Sigma-Aldrich), 1% l-glutamine and 10% heat-inactivated fetal bovine serum (FBS, Gibco) and stimulated with the T cell mitogen ConA (Sigma-Aldrich) at 5 μg/mL for 72 h in a humidified incubator 37 °C/5% CO_2_. Cells were lysed by the addition of 1 mL of cell lysis buffer containing 0.1% v/v beta-mercaptoethanol. Total RNA was isolated using the RNeasy Mini Kit (Qiagen Inc., Manchester, UK) following the manufacturer’s instructions and cDNA was prepared by reverse transcription using SuperScript^®^ III Reverse Transcriptase (Invitrogen, Life Technologies, Paisley, UK) to serve as a template for the cloning of the *IL*-*17A* genes. The gene encoding bovine (bov) IL-17A was amplified using specific primers encoding the full length sequences (for cattle bovIL17apEExsF2: CAA TAA GCT TCC ATG GCT TCT ATG AGA ACT TC and bovIL17apEExsR3: TCT GCC CGG GTC TTA AGC CAA ATG GCG) flanked by restriction enzymes sites *Hin*dIII and *Sma*I to facilitate sub-cloning into pEE14 expression vector (Lonza, Slough, UK). At the start of the study no ovine IL-17A sequence was available so primers based on the bovine and caprine IL-17A sequences were used to clone the ovine gene (cahIL17apEExsF2: CAA TAA GCT TCC ATG GCG TCT ATG AGA ACT GC and ovIL17xsR2: TCT GCC CGG GTC TTA AGC CAC ATG GCG GAC) along with the restriction enzymes sites *Hin*dIII and *Sma*I to facilitate sub-cloning. BovIL-17A cDNA sequence derived from ConA-stimulated PBMC was identical to the previously reported sequence (NM_001008412.2). The sequence of ovIL-*17A* derived in this study (LN835312, European Nucleotide Archive record) has a 100% identity with the *ovIL*-*17A* (XP_004018936.1) predicted from genomic DNA.

Conventional PCR protocols were undertaken to amplify the full length genes in a reaction containing: 1 µL of cDNA, 2.5 µL of 10× PCR buffer, 1.5 µL of MgCl_2_, 0.5 µL 10 mM dNTP, 0.1 µL of a mix of 10:1 Taq DNA polymerase (5 U/mL) (Bioline, UK) and Pfu DNA polymerase (5 U/mL) (Promega, Madison, USA) and PCR water (Sigma-Aldrich) to a volume of 25 μL. The PCR conditions for the amplification of both bovIL-17A and ovIL-17A consisted of an initial denaturation of 5 min at 95 °C, followed by 40 cycles of 94 °C for 30 s, 60 °C for 30 s and 72 °C for 1 min. The PCR products were visualised on a 1% w/v agarose gel containing SYBR^®^ Safe DNA gel stain (Invitrogen, Life Technologies) using a UV light box and purified using a QIAquick Gel Extraction Kit (Qiagen Inc.) before ligation into pGEM-T Easy Cloning Vector (Promega).

After the transformation into XL1-Blue Competent Cells (Stratagene, Agilent Technologies Division, USA), the cells were grown on Luria–Bertani (LB) agar (Sigma-Aldrich) supplemented with X-Gal and 10 mM IPTG overnight at 37 °C. White colonies were selected and grown overnight in 5 mL of LB medium with ampicillin (100 µg/mL, Sigma-Aldrich), in a shaking incubator at 37 °C. Plasmid DNA from four independent colonies of bovIL-17A and ovIL-17A cDNAs was purified using a QIAprep Plasmid DNA Miniprep kit (Qiagen Inc.) following the manufacturer’s instructions and then sequenced to confirm the full length sequences using the T7 and SP6 sequencing primers (Eurofins Genomics, Ebersberg, Germany).

Bovine IL-17A and ovIL-17A cDNAs were compared for similarity using the Basic Local Alignment Search Tool (BLAST 2.5.1, [16, 17]). The predicted amino acid sequences were then analysed for the presence of a signal peptide using Signal 4.1 [18, 19]. The mature protein sequences were aligned with the corresponding sequences from a variety of vertebrates including representative mammal, reptile and avian species using Clustal Omega [20, 21]). A matrix of pair-wise identity at the amino acid level was generated using Clustal 2.1. Evolutionary sequence comparisons were undertaken using 13 selected mammalian and other sequences with the multiple alignment generated using Clustal Omega. Prior to running the phylogenetic analysis the most appropriate amino acid substitution model was obtained by running the model selection module of TOPALi v2.5 [22]. The evolutionary relationships between the sequences were inferred using Mr. Bayes launched from TOPALI v2.5 using the Jones–Taylor–Thornton plus gamma (JTT + G) model with two runs each of 1 250 000 generations with a burn in period of 20% and sampling frequency of 1000.

### Expression vector construct production

The pEE14 vector was linearized and the confirmed bovIL-17A/ovIL-17A excised from pGEM-T Easy clones by double digestion using *Hin*dIII and *Sma*I at 37 °C for 3 h in 30 µL reactions. The digests were run in a 1% w/v agarose gel and bands excised and purified with a QIAquick Gel Extraction kit (Qiagen Inc.). The cDNA and linearized expression vector were then ligated in 1:3 ratio of insert/vector using T4 DNA ligase (Promega) at 4 °C overnight. Ligated products were transformed into competent JM109 cells (Promega), and seeded into LB plates with ampicillin (100 µg/mL). Positive colonies were grown overnight in 5 mL of LB medium with ampicillin in a shaking incubator at 37 °C. Plasmid DNA was purified as above and quantified using the Nanodrop spectrophotometer (NanoDrop Technologies, Thermo Fisher Scientific, MA, USA). Plasmid constructs were sequenced (Eurofins) to verify the integrity of the bovIL-17A and ovIL-17A sequences.

### Expression and quantification of recombinant bovine IL-17A and ovine IL-17A

Chinese Hamster Ovary (CHO) cells were used as the expression system for the cloned cytokine cDNAs. The CHO cells were maintained in Glasgow’s modified Dulbecco’s Medium (GMEM, Sigma-Aldrich) supplemented with minimum essential medium non-specific amino acids (Gibco), 1 mM sodium pyruvate (Gibco), 410 μM glutamic acid (Sigma-Aldrich) with 450 μM l-asparagine (Sigma-Aldrich), 1.3 mM adenosine (Sigma-Aldrich), nucleosides (1.2 mM guanosine, 1.4 mM cytidine, 1.4 mM uridine and 495 μM thymidine, all Sigma-Aldrich), 10% heat-inactivated FBS (PAA Gold, Little Chalfont, UK) and 2 mM l-glutamine (Sigma-Aldrich), designated parent CHO medium [23]. CHO cells were subcultured twice weekly in 75 cm^2^ vent-capped tissue culture flasks (Corning Costar, Scientific Laboratory Supplies Ltd) using 0.05% trypsin in versene/EDTA (Gibco and Sigma-Aldrich) for detachment. For transfection, cells were seeded at 3 × 10^5^ cells/well in a six well plate (Nunc, Roskilde, Denmark) and incubated overnight at 37 °C/5% CO_2_. Cells were then transfected with 3 µg of the plasmid vector pEE14 containing cDNA encoding either bovIL-17A or ovIL-17A cDNA using Lipofectamine 2000 (Invitrogen, Life Technologies Ltd) according to the manufacturer’s instructions. Transfected CHO cells were initially established in transfectant CHO medium comprising GMEM containing 7.5% dialysed (d) glutamine-free heat-inactivated FBS (Invitrogen) and 25 µM methionine sulfoximine (MSX; Sigma-Aldrich).

Cells that survived the selective MSX inhibitor were subsequently amplified with increasing concentrations of MSX prior to cloning by limiting dilution to establish stable, cloned transfectant cell lines as previously described [24]. The supernatants from the IL-17A-transfected CHO cells were tested for the presence of rIL-17A using a commercial bovine IL-17A ELISA with quantifiable reference standards (Kingfisher Biotech, Minneapolis, USA).

### Validation of transfected CHO cells expressing recombinant bovine IFN-γ and ovine IFN-γ

Existing transfected CHO cell lines expressing rbovIFN-γ and rovIFN-γ were used as positive controls for detection of intracellular IFN-γ. The CHO-expressed rbovIFN-γ and rovIFN-γ were evaluated by an in-house ELISA using the anti-bovine IFN-γ mab clones CC330 and CC302 (Bio-Rad Laboratories, Oxford, UK) and commercial rbovIFN-γ (Pierce Endogen, Rockford, USA) as a quantifiable reference standard. The biological activities of the rbovIFN-γ and rovIFN-γ were confirmed using a viral inhibition bioassay as described by Entrican et al. [25].

### Bulk recombinant cytokine production and functional determination of recombinant bovine and ovine IL-17A

For bulk recombinant cytokine production and matched negative-control supernatant, transfected and parent CHO cell lines were maintained routinely in 225 cm^2^ flasks and sub-cultured twice weekly at a 1:10 ratio as previously described. Multiple flasks with sub-confluent cell monolayers from each cell line were grown prior to the preparation of serum-free conditioned medium as previously described [24], clarified by centrifugation at 1000* g* at 4 °C for 10 min and stored at −80 °C until required. The CHO-expressed rbov and rovIL-17A were tested for their capacity to stimulate CXCL8 production in vitro using an Embryonic Bovine Lung cell line (EBL, kindly provided by Dr. Amin Tahoun and Professor David Gally, RI) and the ovine ST-6 cell line [26]. The EBL cells were subcultured in Dulbecco’s Modified Eagle Medium (DMEM, Invitrogen) containing 10% heat-inactivated FBS (PAA) defined as culture medium, using 75 cm^2^ vent-capped tissue culture flasks (Corning Costar, Scientific Laboratory Supplies Ltd). The ST-6 cells were similarly subcultured in Iscove’s Modified Eagle Medium (IMDM, Gibco, Life Technologies) containing 10% heat-inactivated FBS (PAA). Cells were adjusted to 1 × 10^5^/mL in culture medium and seeded in triplicate, at 500 µL/well in 48 well plates (Corning Costar, Scientific Laboratory Supplies Ltd) then cultured in a humidified incubator at 37 °C/5% CO_2_ overnight. The culture medium was then replaced with either serum-free conditioned CHO medium containing rbov or rovIL-17A adjusted to 100 ng/mL or serum-free conditioned medium from untransfected CHO cells at an equivalent dilution. The resultant supernatants from the treated EBL and ST-6 cells were harvested 24 h later and stored at −20 °C until analysis for the presence of CXCL8 by ELISA. This ELISA protocol has been described elsewhere in detail [27] and is based on a murine anti-ovine CXCL8 mab 8M6 as a capture and rabbit anti-sheep CXCL8 polyclonal antibody for detection (Bio-Rad Laboratories). This ELISA has also been shown to detect native bovine CXCL8 [28, 29].

### Generation of native ovine IL-17A

PBMC from six ewes (2–4 year old Greyface breed) were cultured at 2 × 10^6^ cell/mL in 100 μL of IMDM supplemented with 10% heat-inactivated FBS, 50 μg/mL gentamicin and 50 μM 2-mercaptoethanol in the presence or absence of 100 μL per well ConA (5 μg/mL) in a 96 well U-bottom plate (Nunc) in a humidified incubator at 37 °C/5% CO_2_. Quadruplicate wells were set up for each treatment for the six animals. The culture supernatants from the technical replicates were harvested and pooled together after 96 h and stored at −20 °C prior to analysis by the commercial anti-bovine IL-17A ELISA (Kingfisher Biotech).

### IL-17A ELISpot

MultiScreen-IP Filter Plates (Merck Millipore, Hertfordshire, UK) were activated by addition of 70% ethanol (Fisher Scientific, Loughborough, UK) for a maximum of 2 min. Plates were washed five times with sterile ddH_2_O and then incubated with 50 µL/well of 5 µg/mL rabbit polyclonal anti-bovine IL-17A antibody (product code PB0274B-100, Kingfisher Biotech) for 18 h at 4 °C. Simultaneously, PBMC from three cattle (8–12 month old Holstein–Friesian) and three ewes (3 year old Texel-cross) were re-suspended in RPMI 1640 medium containing 10% heat-inactivated FBS, 2 mM l-glutamine, 100 U/mL penicillin, 100 μg/mL streptomycin and 50 μM 2-mercaptoethanol (RPMI culture medium) at a concentration of 2 × 10^6^ cells/mL and stimulated with either 5 µg/mL ConA or culture medium alone for 18 h or for the last treatment culture medium for 12 h replaced with 50 ng/mL phorbol 12-myristate 13-acetate (PMA, Sigma-Aldrich) and 1 µg/mL ionomycin (ionomycin calcium salt from *Streptomyces conglobatus*, Sigma-Aldrich) for the final 6 h in a humidified incubator at 37 °C/5% CO_2_. The plates were set up for three technical replicates for each treatment for each animal.

Following incubation with the coating antibody, the ELISpot filter plate was washed five times with sterile PBS and subsequently incubated with 100 µL/well RPMI culture medium for 30 min at RT. Medium was removed and 2 × 10^5^ stimulated or unstimulated cells were added to each well, with each treatment in triplicate wells for each animal. The plate was incubated overnight at 37 °C/5% CO_2_. The cells were removed from the ELISpot plate wells and discarded. The plate was washed five times with PBS and 0.5 µg/mL biotinylated rabbit polyclonal anti-bovine IL-17A antibody (product code number PB0277B-50, Kingfisher Biotech) in PBS with 0.5% FBS was added to the wells and incubated for 2 h at RT. After a further five washes with PBS, wells were incubated with a 1:1000 dilution of streptavidin conjugated to horse radish peroxidase (Sigma-Aldrich) in PBS with 0.5% FBS for 1 h at RT. After a final five washes with PBS, wells were incubated with SureBlue™ TMB Microwell Peroxidase Substrate (KPL, Maryland, USA) for 10 min at RT. Reactions were stopped by washing wells five times with ddH_2_O and the plate was left to dry overnight prior to analysis using an AID EliSpot Reader HR (AID Autoimmun Diagnostika GmbH, Straßberg, Germany) using AID EliSpot software version 4.0.

### Evaluation of commercial antibodies for intracellular detection of IL-17A

The cloned, transfected CHO cells served as source of constitutive IL-17A expression for evaluation of antibodies and the CHO parent cells served as negative control cells. CHO cells were cultured as described above. For flow cytometry, cells were pelleted by centrifugation at 626 *g* at 4 °C for 2 min and washed three times in cold PBS before being fixed in 0.5 mL of 1% paraformaldehyde (PFA, Sigma-Aldrich) at RT for 10 min. Cells were washed once more in cold PBS and adjusted to 1 × 10^7^ cells/mL in PBS/0.05% (w/v) sodium azide (Sigma-Aldrich) and stored at 4 °C. The transfected CHO cells were prepared in advance and have been shown to be stable (without significant changes to detection of recombinant proteins by ICS or cell autofluorescence) when stored at +4 °C for several weeks (data not shown). Transfectant CHO cells stored in this way have been used to evaluate commercial antibodies for their capacity to bind recombinant ovine TNF-α [30] and recombinant ovine FoxP3 [31]. Prior to staining the cells underwent an overnight permeabilisation and block step. Cells were pelleted by centrifugation at 626* g* for 2 min at 4 °C then resuspended to 10^7^ cells/mL in PBS containing 5% heat-inactivated FBS (PAA Gold), 0.05% (w/v) NaN_3_ (Sigma-Aldrich), 0.2% (w/v) saponin (Sigma-Aldrich) (permeabilisation buffer) plus 20% normal goat serum (Merck Millipore) (combined permeabilisation and blocking buffer) and maintained overnight at 4 °C consistent with our established protocol using transfectant CHO cells [30, 31].

The permeabilised and blocked CHO cells were then transferred to 96 well U-bottom plate (BD Falcon, Massachusetts, USA), 50 µL of cell suspension per well per labelling antibody. The cells were pelleted by centrifugation at 626* g* for 2 min at 4 °C, aspirated in permeabilisation buffer and then 100 µL primary antibody or isotype/equivalent matched control antibody or permeabilisation buffer alone was added for 30 min at 4 °C. A full detailed list of the commercial anti-IL-17A antibodies used in this study is provided in Table 1 and these were used according to the manufacturer’s recommendations. A list of isotype-matched or equivalent polyclonal antibodies used as appropriate negative controls is shown in detail in Table 2. Cells were then washed by centrifugation at 626 *g* for 2 min at 4 °C three times in permeabilisation buffer. The secondary antibody consisting of 100 μL per well goat anti-mouse-IgG-phycoerythrin (PE) conjugated (Invitrogen, USA) at 1 μg/mL in permeabilisation buffer for cells labelled with the primary mabs or 100 μL per well goat anti-rabbit IgG alexafluor 488 at 1 μg/mL for cells labelled with the primary pabs. Cells labelled using the RPE-conjugated anti-IL-17A mab were incubated in 100 μL per well in permeabilisation buffer alone for 30 min at 4 °C. Cells were washed twice in permeabilisation buffer then finally washed in PBS prior to fixation in 200 μL of 1% PFA and stored at 4 °C in the dark prior to acquisition.

**Table 1:** **Commercial anti-IL-17A antibodies evaluated by intracellular staining for capacity to bind recombinant bovine and ovine IL-17A**

Primary antibody used in the intracellular staining (CHO cells)	Clone	Commercial supplier and product code in brackets	Antibody isotype	Immunogen	Specificity	Antibody conjugation if present
A.1	N/A rabbit derived polyclonal antibody. Lot number BO1431JK	Kingfisher Biotech (PB0274B-100)	IgG1	Recombinant bovine IL-17A	Bovine	None
B.1	eBio64Dec17	eBiosciences (12-7179-41)	IgG1	Recombinant human IL-17A	Human	PE
C.1	MT44.6	Mabtech (3520-3-250)	IgG1	Recombinant human IL-17A	Human, rhesus and cynomolgus macaques and common marmoset monkeys	None
C.2	MT241	Mabtech (3520M-3-250)	IgG1	Recombinant human IL-17A	Human, rhesus and cynomolgus macaques	None
C.3	MT2770	Mabtech (3521-14-250)	IgG1	Recombinant mouse IL-17A	Mouse	None
C.4	MT504	Mabtech (3520-6-250)	IgG1	Recombinant human IL-17A	Human, rhesus and cynomolgus macaques and common marmoset monkeys	Biotin
D.1	41809	R & D Systems IC317P	IgG2b	Recombinant human IL-17A amino acids 20-155	Human	PE
D.2	41802	R & D Systems (IC3171P)	IgG1	Recombinant human IL-17A amino acids 20-155	Human	PE

All details of antibody clone, product code, immunogen, host specificity and antibody conjugate have been taken from supplier datasheets. Each antibody has been assigned a code where the capital letter denotes the commercial supplier and the integer an individual pab or a mab clone. The commercial antibodies were used in conjunction with an appropriate control antibody (see Table 2, denoted by the equivalent letter but in lowercase) for the intracellular staining protocol described in “Evaluation of commercial antibodies section”.

**Table 2:** **Isotype control mabs and control pab used in the evaluation of the commercial anti-IL-17A antibodies**

Control antibody used in the intracellular staining (CHO cells)	Clone	Source	Immunogen/host raised in	Isotype	Antibody conjugation, if present
a	N/A	Professor Waithaka Mwangi, A & M University, Texas, USA	Bovine CD34 construct/rabbit	Rabbit IgG	None
b, c and d	VPM 21	Moredun	Border disease virus/mouse	Mouse IgG1	None
d	VPM22	Moredun	Border disease virus/mouse	Mouse IgG2b	None

The two mabs VPM21 and VPM22 (both MRI, Edinburgh, UK) and control pab (raised against bovine CD34; A & M University, Texas, USA) were derived from non-commercial sources. The hybridoma cell lines were grown in-house to generate mabs that were then adjusted for concentration to match that of the anti-IL-17A mabs listed in Table 1. The antibodies were assigned a code letter in lower case that corresponds with the commercial antibodies listed in Table 1 (denoted by the equivalent uppercase letter) for the intracellular staining protocol followed in “Evaluation of commercial antibodies section”.

Cells were acquired for flow cytometric analyses using the MacsQuant flow cytometer (Miltenyi Biotech, Germany) and analysed using the MacsQuantify Software v2.7. 20 000–50 000 events were collected and the subsequent gating strategy shown in Additional file 2 followed. Briefly, after removing artefacts with gating P1 (Additional file 2A), P1/P2 was applied to the main population to exclude debris (Additional file 2B) followed by P1/P2/P3 for doublet discrimination (Additional file 2C). The positive threshold setting in the phycoerythrin or alexafluor 488 channels P1/P2/P3/P4 were set using the isotype or equivalent control for each CHO cell line (Additional file 2D). Overlaying histogram plots of P1/P2/P3 were used to compare anti-IL-17A antibodies with appropriate isotype or equivalent controls (Additional file 2E). Gated percentage numbers (P1/P2/P3/P4) and median fluorescence region values (P1/P2/P3) were measured for each antibody. Delta median fluorescence intensity (deltaMFI) was calculated by deducting the median mab isotype or pab control median fluorescence region value from the anti-IL-17A antibody median fluorescence region value. The summarised data are presented in Additional file 3.

### IL-17A and IFN-γ expression by bovine and ovine T cell subsets

PBMC were prepared from whole blood from four cattle (2 year old female Holstein–Friesian) and four sheep (3 year old Texel-cross), resuspended in RPMI-1640 culture medium, counted using Trypan Blue (ThermoFisher Scientific, Utah, USA) and adjusted to 1 × 10^7^ cells/mL. 2 × 10^7^ cells were stimulated with PMA (50 ng/mL), ionomycin (1 µg/mL), brefeldin A (10 µg/mL) diluted in RPMI culture medium for 4 h in sterile centrifuge tubes in a humidified incubator at 37 °C/5% CO_2_.

Cells were pelleted by centrifugation at 258* g* for 5 min at 4 °C and washed in PBS and samples reserved as controls for the live/dead stain for flow cytometric analyses. 1 × 10^7^ cells were stained using the violet live/dead Fixable Dead Cell Stain Kit (Life Technologies) according to the manufacturer’s protocol. Cells were fixed in 1% PFA for 10 min at RT. PBMC were permeabilised overnight as described above for the CHO cells (which also served as positive controls for the PBMC intracellular cytokine staining). 1 × 10^6^ permeabilised PBMC were transferred into each well of a 96 well U-bottom plate (BD Falcon, Massachusetts, USA) then pelleted by centrifugation. A combined cell surface phenotyping and intracellular cytokine staining step was undertaken with directly-conjugated mabs and the appropriate isotype-matched and FMO controls in a volume of 100 μL per well for 30 min at RT (Table 3). Cells were pelleted, washed once in permeabilisation buffer and then once in PBS before final resuspension in 150 μL PBS. A minimum of 50 000 events were acquired using an LSRFortessa™ cell analyzer (Becton–Dickinson) and analysed using FlowJo vX for Windows 7 using the gating strategies shown in Additional file 4 (cattle cells) and Additional file 5 (sheep cells).

**Table 3:** **Commercial antibodies used in the detection of native intracellular IL-17A and IFN-γ by bovine and ovine T cell subsets**

Antibody clone	Commercial supplier and product code in brackets	Antibody isotype	Target antigen	Host specificity	Antibody conjugation	Antibody dilution
CC8	Bio-Rad Laboratories (MCA1653PE)	IgG2a	CD4	Bovine	Phycoerythrin	1:20
44.38	Bio-Rad Laboratories (MCA2213PE)	IgG2a	CD4	Ovine	Phycoerythrin	1:20
CC58	Bio-Rad Laboratories (MCA1654PE)	IgG1	CD8β	Bovine	Phycoerythrin	1:20
CC15	Bio-Rad Laboratories (MCA838PE)	IgG2a	WC-1	Bovine	Phycoerythrin	1:200
CC302	Bio-Rad Laboratories (MCA1783A647)	IgG1	IFN-γ	Bovine	Alexafluor-647	1:200
eBio64DEC17	eBiosciences (17-7179)	IgG1	IL-17A	Human	APC	1:20
F8-11-13	Bio-Rad Laboratories (MCA1209PE)	IgG1	Recognised rat cell surface marker	Rat	Phycoerythrin	1:20
MRC OX-34	Bio-Rad Laboratories (MCA929PE)	IgG2a	Rat cell surface marker	Rat	Phycoerythrin	1:20

All details of antibody clones, product code, immunogen, host specificity and antibody conjugate are taken from datasheets provided by the commercial suppliers. The antibodies were used at the described dilutions for the cell phenotyping and intracellular cytokine staining described in “Expression of intracellular IL-17A and IFN-γ by bovine and ovine T cell subsets section”.

### Statistical analyses

The various datasets were analysed separately for statistical interpretation and the methods used will be outlined sequentially. The IL-17A bioassay datasets for the differential CXCL8 expression in technical replicates stimulated with rbovIL-17A, rovIL-17A and CHO UTF control supernatant was statistically assessed using Kruskal–Wallis tests for both bovine (EBL) and ovine (ST-6) cell lines. The native IL-17A expression of ovine PBMC from six animals in re-recall assays with ConA was analysed using the two-tailed Mann–Whitney test.

The ELISpot data were modelled by fitting a Poisson generalised linear mixed model (GLMM) by maximum likelihood to the IL-17A SFU/10^6^ production, using logarithmic link function and Laplace approximations to calculate log-likelihoods. The model included treatment (ConA, medium and PMA/ionomycin), species (bovine, ovine) and their interaction as fixed effects and animal identification as a random effect in order to account for both within- and between-animal variability. An observation-level random effect term was specified to account for data over-dispersion. The statistical significance of the fixed effect terms was assessed using *p* values derived from type II Wald Chi square tests. Linear hypothesis tests were defined from the GLMM in order to conduct pair-wise comparisons of means between treatments and species. The associated *p* values were adjusted for false discovery rate (FDR) following Benjamini–Hochberg’s procedure [32].

A principal component analysis (PCA) was conducted to investigate the overall relationships between commercial antibodies and specific binding capacity to rbov and rovIL-17A on the basis of the six metrics considered simultaneously. This statistical technique projects the information in multi-variable/-parameter datasets onto low dimensions, typically two dimensions, which is useful here to facilitate meaningful comparisons and clustering of the commercial antibodies by means of optimal linear combinations (principal components, PCs) of the original metrics, that are represented visually in a biplot [33, 34]. For example, Hemmink et al. [35] have used PCA to analyse immunological datasets following an influenza pathogenesis study in pigs showing the correlation of cytokine production, with viral titre over time. Our antibody screening results were displayed using a biplot based on the two first PCs (those accounting for the highest percentage of the total observed data variability) to facilitate discussion and ranking of the commercial antibodies. Finally, in the bovine and ovine T cell subset phenotyping and combined intracellular staining datasets, the total percentage IL-17A and IFN-γ expression for cattle and sheep PBMC were used to statistically assess the significance of differences in expression between species using two-tailed Mann–Whitney tests allowing for ties.

Statistical test significance was assessed at the 5% significance level. The statistical analyses were conducted on the R system for statistical computing v3.2.

## Results

### Genetic relationships of mammalian IL-17A sequences

**Figure 1:**
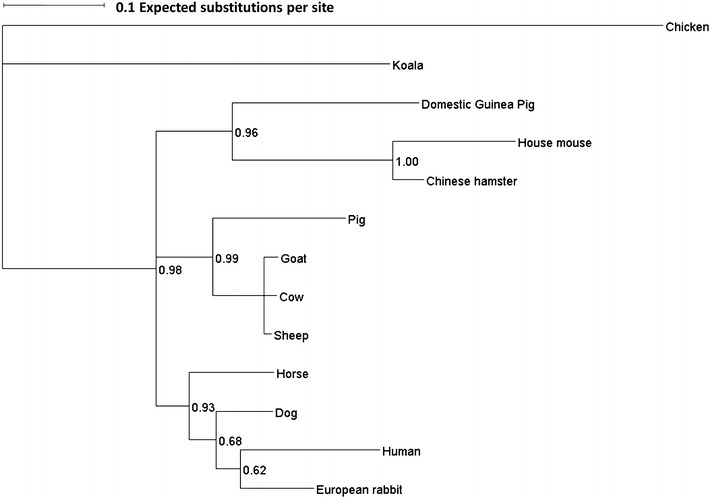
**Phylogenetic tree of mammalian IL-17A protein sequences.** Evolutionary sequence comparisons were undertaken using 13 selected mammalian and other IL-17A sequences by initially conducting a multiple alignment using Clustal Omega (EMBL/EBI online, [21]). The evolutionary relationships between the sequences were inferred using Mr. Bayes launched from TOPALI v 2.5 using the Jones–Taylor–Thornton plus gamma (JTT + G) model with two runs each of 1 250 000 generations with a burn in period of 20% and sampling frequency of 1000. The horizontal lines are branches whose length represents the amount of genetic change over time. The scale bar shows the distance represented by 0.1 expected substitutions per site. The robustness of the clustering of sequences are shown by the Bayesian Posterior Probabilities at the nodes. Accession numbers of the sequences used for the comparison are: Human NP_002181.1; House mouse NP_034682.1; Cow NP_001008412.1; Sheep XP_004018936.1; Goat NP_001272654.1; Horse NP_001137264.1; Pig NP_001005729.1; Dog NP_001159350.1; Domestic guinea pig NP_001265697.1; Koala AHZ08738.1; Chicken NP_989791.1; EGW10039.1 Chinese hamster and European rabbit AMQ91106.1. The phylogenetic tree was annotated using Dendroscope.

BovIL-17A and ovIL-17A cDNA coding sequences were 462 nucleotides in length and shared 97% identity. The predicted amino acid sequences including the signal peptide corresponded to 153 amino acids. According to SignalP 4.1, both bovIL-17A and ovIL-17A signal peptides account for the first 23 amino acids and the mature peptides are 130 amino acids in length. The pair-wise identity matrix of the bovIL-17A and ovIL-17A with other published mature peptide sequences revealed identities of between 48.4 and 89.5% to the chicken and wild Bactrian camel respectively (Additional file 6). Further analysis revealed over 99% identity between the bovine, ovine and caprine IL-17A amino acid sequences and a high degree of identity to human IL-17A (bovine and ovine 76.2%; caprine 75.8%). Evolutionary sequence comparisons using 13 selected mammalian and other sequences were inferred using Mr. Bayes launched from TOPALI v 2.5 using the Jones–Taylor–Thornton plus gamma (JTT + G) model, as shown in Figure 1. The sequences cluster to three groups with the caprine, bovine, ovine and swine sequences in one cluster. Given the pair-wise identities and the evolutionary relationships between the human and bovine and ovine sequences, we speculated that there would be a strong likelihood of mabs produced against human IL-17A binding to the bovine and ovine orthologues and that this could be definitively determined using the mammalian CHO expression system.

### Expression, detection and biological function of recombinant ruminant IL-17A

**Figure 2:**
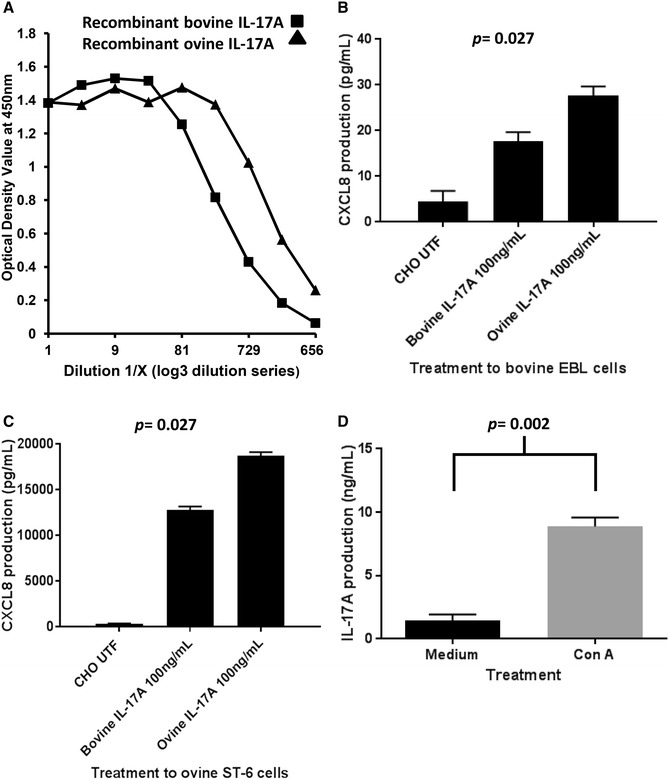
**Measurement and biological function of recombinant bovine and ovine IL-17A and detection of native ovine IL-17A by ELISA. A** Detection of rbov and rovIL-17A by ELISA. The supernatants from transfected CHO cells expressing rbovIL-17A or rovIL-17A, or control parent untransfected line (UTF) were serially diluted (Log_3_ dilutions) and evaluated using the commercial bovIL-17A ELISA. Data presented are optical density (OD) values from the Spectrophotometer at 450 nm. The X-axis displays Dilution 1/X and the Y-axis gives the OD value. Readings from UTF supernatant were below the limit of detection. **B** Functional activity of rbov and rovIL-17A on bovine embryonic lung cells. Bovine embryonic lung (EBL) cells were stimulated with 100 ng/mL CHO-expressed rbovIL-17A or rovIL-17A or UTF CHO negative control supernatant. Following 24 h incubation, culture supernatants were collected from triplicate cultures then tested for CXCL8 by ELISA. The X-axis displays the bioassay treatments and the Y-axis shows CXCL8 production in pg/mL. Data are the arithmetic mean of three technical replicates with error bars representing the standard error from one of three experiments. CXCL8 expression between treatments was statistically assessed using Kruskal–Wallis test. **C** Functional activity of rbov and rovIL-17A on ovine ST-6 cells. Ovine ST-6 cells were stimulated with 100 ng/mL CHO-expressed rbovIL-17A or rovIL-17A or UTF CHO supernatant. Following 24 h incubation and culture supernatants collected, tested and analysed as described in Figure 2B. CXCL8 expression between treatments was statistically assessed using Kruskal–Wallis test. **D** Detection of native ovIL-17A by ELISA. Ovine PBMC were cultured at 2 × 10^6^ cells/mL with or without 5 μg/mL ConA. Culture supernatants were analysed for IL-17A using the bovIL-17A ELISA. Data represent the arithmetic mean of PBMC from six ewes and error bars represent standard error. Data were analysed statistically for significance using the two-tailed Mann–Whitney test.

The Kingfisher Biotech bovine IL-17A VetSet is a pab-based ELISA for detection of bovine IL-17A and based on the homology between the bovine and ovine orthologues, we predicted that it would detect both of the expressed recombinant proteins. Figure 2A shows titration curves for supernatant from transfected CHO cells expressing rbovIL-17A and rovIL-17A demonstrating the dynamic range that the rbov and rovIL-17A can be detected at using this commercial ELISA test. No signal was detected in supernatant from untransfected CHO cells (data not shown). The supernatant from the CHO line expressing rovIL-17A titrated over a wider range of concentrations than the rbovIL-17A-expressing CHO line. Quantification of the supernatants using the Vetset kit recombinant bovine IL-17A standard revealed that rovIL-17A and rbovIL-17A supernatants had concentrations of 8809 and 1660 ng/mL respectively.

We next established that the expressed recombinant proteins were functionally active. The supernatants were adjusted to 100 ng/mL for direct comparison of their ability to stimulate CXCL8 expression in a bovine (EBL) and ovine (ST-6) cell line (Figures 2B and C respectively). The magnitude of CXCL8 production by the two cell lines was notably different, with higher levels observed in the ovine ST-6 cell line compared to the bovine EBL cell line, irrespective of whether they were stimulated with rbovIL-17A or rovIL-17A. One effect of rIL-17A is shown by the mean up-regulation of CXCL8 over the background levels produced by EBL cells (*p* = 0.027) or ST-6 cells (*p* = 0.027) exposed to supernatant from untransfected CHO cells. The rovIL-17A stimulated more CXCL8 production than rbovIL-17A in both cell types. Having shown that the bovine IL-17A Vetset ELISA detects rovIL-17A, consistent with publically-available cross-species reactivity information, it was important to confirm that it could also detect and quantify native ovine IL-17A. Culture supernatants from ovine PBMC incubated for 96 h in the presence or absence of ConA were assessed for the presence of IL-17A by the Vetset ELISA. A low level of endogenous IL-17A was found in unstimulated cells, which was statistically significantly upregulated in response to ConA stimulation (*p* = 0.002, Figure 2D).

### Enumeration of ruminant cells expressing IL-17A by ELISpot

**Figure 3:**
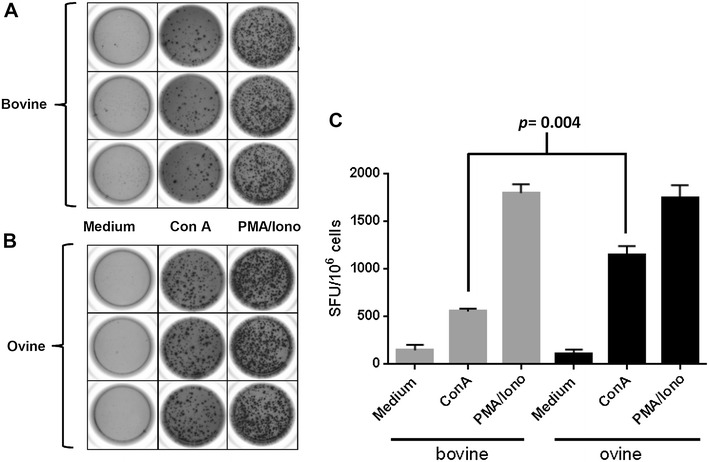
**Detection of single-cell expression of ruminant IL-17A by ELISpot.** Plates and PBMC were prepared and cultured as described in “IL-17A ELISpot section”. ELISpot images shown are representative of PBMC from one of three cattle (**A**) and one of three sheep (**B**) activated with ConA and PMA/ionomycin. The average number of spot-forming units (SFU) with standard errors are shown for 10^6^ PBMC from all three cattle (grey bars) and sheep (black bars), stimulated under the different conditions (**C**). Data were modelled by fitting a Poisson generalised linear mixed model (GLMM) by maximum likelihood to the IL-17A SFU/10^6^ values, using logarithmic link function and Laplace approximations to calculate log-likelihoods. The model included treatment (medium control, ConA and PMA/ionomycin), species (bovine, ovine) and their interaction as fixed effects and animal identification as a random effect in order to account for both within- and between-animal variability. An observation-level random effect term was specified to account for data over-dispersion. The statistical significance of the fixed effect terms was assessed using *p* values derived from type II Wald Chi square tests. Linear hypothesis tests were defined from the GLMM in order to conduct pair-wise comparisons of means between treatments and species. The associated *p* values were adjusted for false discovery rate (FDR) following Benjamini–Hochberg’s procedure.

Quantification of cytokines in cell culture supernatants is one method for characterising the polarisation of cellular immune responses and another is the enumeration of cells expressing individual cytokines. Given the ELISA results above, we decided to use the pabs from the Vetset ELISA to develop an IL-17A ELISpot to determine the frequency of IL-17A-producing cells in activated PBMC from cattle and sheep. Figures 3A and B show three technical replicates of PBMC from one cow and one sheep stimulated by ConA or PMA/ionomycin. Consistency of the technical replicates was clearly visible, as was the almost complete absence of IL-17A-secreting cells in the unstimulated controls. Note that these cells were only cultured for 18 h as opposed to 96 h for the ELISA analyses where a positive signal was detected in supernatants of unstimulated cells. Figure 3C shows the average number of spots from activated PBMC from three cattle and three sheep. The average number of spots in cattle and sheep PBMC was similar when activated by PMA/ionomycin [1789.74 Spot Forming Units (SFU) ± 314.42 vs 1731.72 SFU ± 304.29, *p* = 0.895], whereas the mean frequency of IL-17A-producing cells was statistically significantly lower in cattle PBMC compared to sheep PBMC when activated with ConA (546.95 SFU ± 96.32 vs 1130.53 SFU ± 198.76, *p* = 0.004).

### Identification of antibodies that detect recombinant intracellular bovine and ovine IL-17A by flow cytometry

While the ELISpot provides the capability to enumerate the frequency of IL-17A-producing cells and estimates cell-level production determined by spot-size, flow cytometry can provide additional information on the relative levels of IL-17A produced per cell and also identify the phenotype of the producing cell. We used the transfected CHO cells as stable expressers of IL-17A to screen a panel of available mabs and pabs (Table 1) for their ability to detect intracellular bovine and ovine IL-17A. Although the rbovIL-17A and rovIL-17A transfectant CHO cells are derived from the same parent untransfected CHO cells, all three cell lines have different size and granularity properties. To define specific cross-reactive staining of the antibody panel, it was necessary to draw multiple parameter comparisons by overlaying histograms of isotype-matched or appropriate pab controls and calculating the percentages of cells in the gated positive regions from P1/P2/P3/P4 and the deltaMFIs of the entire cell population using the median fluorescence region values within the P1/P2/P3 region relative to appropriate control antibodies (Additional file 2). A summary of the numerical percentage positivity and deltaMFIs for all of the commercial IL-17A antibodies screened against the UTF CHO, rbov IL-17A CHO and rov IL-17A CHO cells can be found in Additional file 3 which provides six measures to evaluate specific capacity to bind the IL-17A cytokine through intracellular staining.

**Figure 4:**
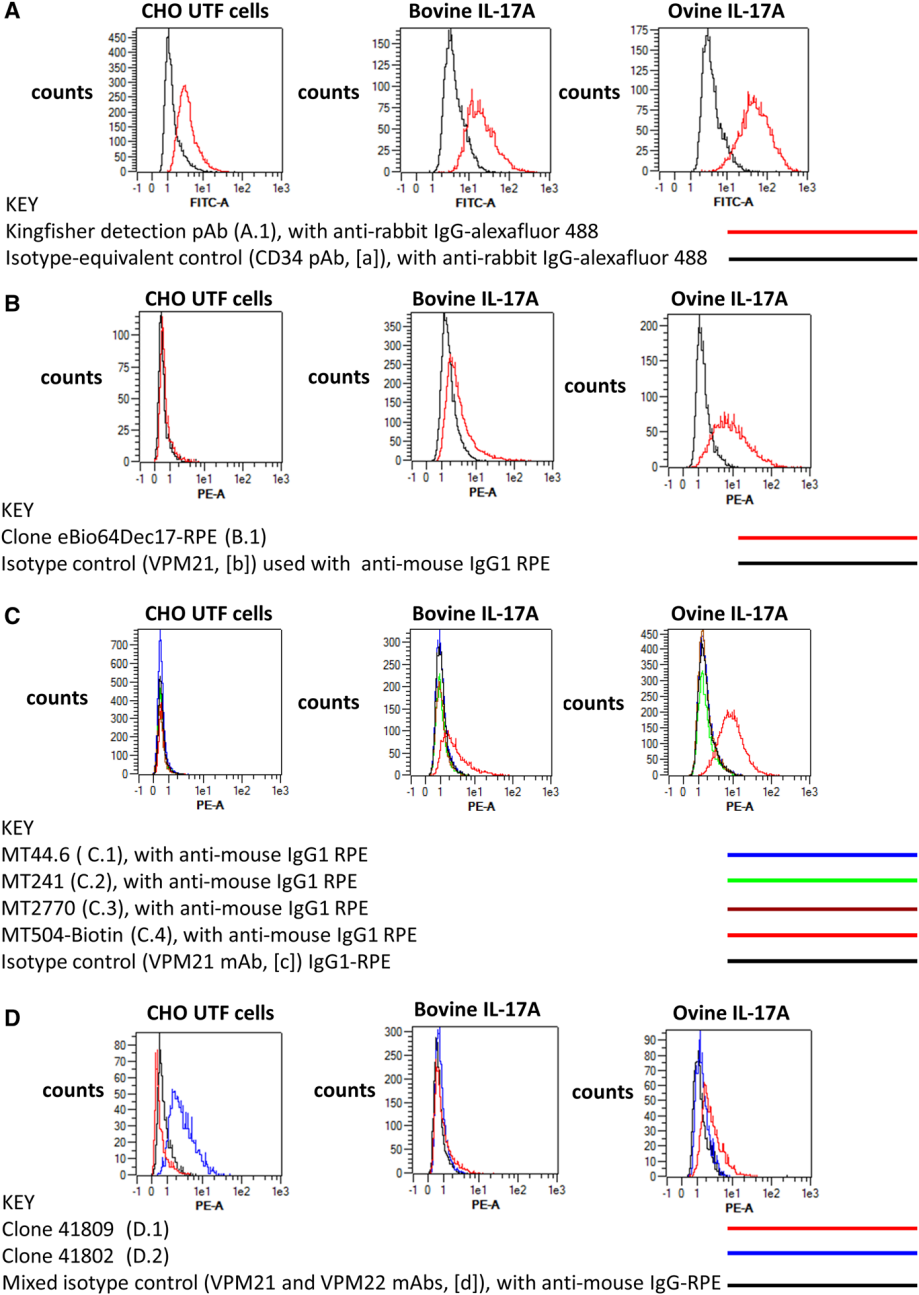
**Evaluation of commercial antibodies for the intracellular detection of recombinant bovine and ovine IL-17A.** The eight commercial antibodies listed in Table 1 were tested against fixed, permeabilised untransfected (UTF) CHO cells and CHO cells transfected with cDNA encoding bovIL-17A or ovIL-17A for their capacity to detect intracellular recombinant IL-17A by flow cytometry. Results are shown for one polyclonal antibody (pab) produced against bovIL-17A (**A**) and seven monoclonal antibodies (mabs) produced against human or mouse IL-17A (**B**–**D**). Profiles of the relevant control antibodies listed in Table 2 are included in the overlapping histograms. Events were acquired on the MacsQuant according to the gating strategy described previously (in brief) and shown in Additional file 2. Line colours representing different antibody treatments are given in parentheses: **A** Primary rabbit anti-bovine IL-17A pab PB0274B-100 at 1 μg/mL (A.1, red) or negative control primary anti-bovine CD34 pab (in-house) at an estimated 1 μg/mL equivalent (a, black) then detected with a secondary goat anti-rabbit alexafluor 488 at 1 μg/mL; **B** Directly conjugated mouse anti-human IL-17A eBio64DEC17-phycoerythrin (PE) mab (IgG1) at 2.5 μg/mL (B.1, red) and control IgG1 VPM21 mab (in-house) at an estimated 2.5 μg/mL equivalent (b, black) and detected with goat anti-mouse PE at 1 μg/mL; **C** Primary mouse anti-human IL-17A mabs MT44.6 (C.1, blue), MT241 (C.2, green), MT2770 (C.3, brown) and MT504 (C.4, red) [all IgG1] at 0.5 μg/mL and control IgG1 VPM21 mab (in-house) at an estimated 0.5 μg/mL equivalent (black), all detected with goat anti-mouse PE at 1 μg/mL; **D** Primary mouse anti-human IL-17A mabs #41809 (D.1, red) (IgG2b) and #41802 (D.2, blue) (IgG1) at 2.5 μg/mL and a mixture of control mabs VPM21 (IgG1) and VPM22 (IgG2b) at an estimated 2.5 μg/mL equivalent (d, black), all detected with goat anti-mouse PE at 1 μg/mL.

As we had previously confirmed that the Vetset pabs detected both recombinant and native bovine and ovine IL-17A by ELISA and ELISpot, we first evaluated the unconjugated pab for intracellular staining of the CHO cells using an anti-rabbit second-stage conjugate. Although positive staining was observed in the transfected CHO cells, there was also a considerable background signal with the untransfected CHO cells, possibly reflective of the use of a pab control antibody (Figure 4A). We then screened the panel of seven mabs (listed in Table 1) against the CHO transfectants. Only two (eBio64DEC17 and MT504) of the seven mabs tested were found to specifically cross-react with rbovIL-17A and rovIL-17A in the CHO cells (Figures 4B and C). Both of these mabs were raised against recombinant human IL-17A. Clone eBio64DEC17 clone gave a higher percentage binding in the positive region and deltaMFI values (38.7%+ve/deltaMFI of 1.03 to the bovIL-17A CHO cells and 92.9%+ve/deltaMFI of 6.07 to the ovIL-17A CHO cells respectively) relative to the isotype-matched mab control (displayed in Figure 4B and metrics shown Additional file 3). The respective values for MT504 were 61.7%+ve/deltaMFI 1.27 and 96.0%+ve/deltaMFI 6.72 (displayed in Figure 4C and metrics shown in Additional file 3). Note the absence of non-specific staining with these mabs on the untransfected control (UTF) CHO cells (displayed in Figures 4B and C). Mab 41809 has a negligible proportion of cells in the positive region whereas mab 41802 gave a high degree of non-specific staining using the UTF CHO cells (deltaMFI of 2.45 and 94.1% of cells in the positive region) compared to the combined isotype controls (Figure 4D). Both mabs have high percentage positivity for bovIL-17A and ovIL-17A based on the region boundary (displayed in Figure 4D and metrics shown Additional file 3), but the very low deltaMFIs (41809: 0.14 (rbov) and 1.12 (rov) and 41802: 0.11 (rbov) and 0.2 (rov) respectively) indicating poor specificity with bovIL-17A and ovIL-17A.

The six metrics used to assess antibody binding to rbov and rovIL-17A (numerical percentage in positive upper region and deltaMFIs for the three CHO cell lines) were represented along with the antibodies in a PCA biplot providing an additional objective way to rank them displayed in Additional file 7. Data for the unconjugated pab were excluded as it was the only non-mab and appeared to be distorting the overall representation of the other antibodies when included in the PCA analyses. The biplot planar representation based on the two first PCs (on the horizontal, PC1, and vertical axes, PC2, respectively) explained 87.37% of the variability of the original data set. The distances between the antibodies (represented by symbol points) spatially reflect their similarity in regards to the original six metrics (represented by axes, with arrows indicating directions of higher values). The mabs eBio64DEC17 and MT504 associate together linking to the highest values of the rbovIL-17A and rovIL-17A deltaMFI variables. The mabs MT44.6, MT241 and MT2270 overlap linking to the lowest values in the rbovIL-17A and rovIL-17A MFI variables. Mab 41809 has a negligible proportion of cells in the positive region whereas Mab 41802 gave a high degree of non-specific staining using the UTF CHO cells (deltaMFI of 2.45 and 94.1% of cells in the positive region) compared to the combined isotype controls (Figure 4D). The angles between axes are representative of the types of correlation between the corresponding metrics. Hence, the small angles observed between the axes for bovIL-17A %, CHO UTF deltaMFI, CHO UTF % and ovIL-17A % on the one hand, and those for ovIL-17A deltaMFI and bovIL-17A deltaMFI on the other hand, are indicative of high correlations within each of these two sets of metrics. The angle of approximately 90° between these two sets of metrics reflects negligible correlation between them. Together our dataset suggests that bovIL-17A and ovIL-17A deltaMFI metrics provide a more reliable measure of specific IL-17A binding than %# bovIL-17A and %# ovIL-17A respectively.

Of all of the antibodies listed in Table 1, only the MT504 mab clone is available in a form that is compatible with use in tissue culture and not stored in a buffer containing sodium azide. As this mab gave positive staining on the transfected CHO cells by flow cytometry, we assessed its potential to neutralise the bioactivity of rbovIL-17A and rovIL-17A. 1 μg/mL of mab MT504 was mixed with 50 ng/mL of rbovIL-17A and rovIL-17A for 2 h at 37 ºC then tested for induction of CXCL8 expression in ovine ST-6 cells as described previously in “Bulk recombinant cytokine production and functional determination of recombinant bovine and ovine IL-17A section” and shown in Figure 2C. The pre-mixing of the mab with recombinant IL-17A resulted in 65.3 and 62.8% decreases in CXCL8 release for rbovIL-17A and rovIL-17A respectively indicating the ability of mab MT504 to neutralise the biological activity of ruminant IL-17A (Additional file 8).

### Expression of intracellular IL-17A and IFN-γ by bovine and ovine T cell subsets

**Figure 5:**
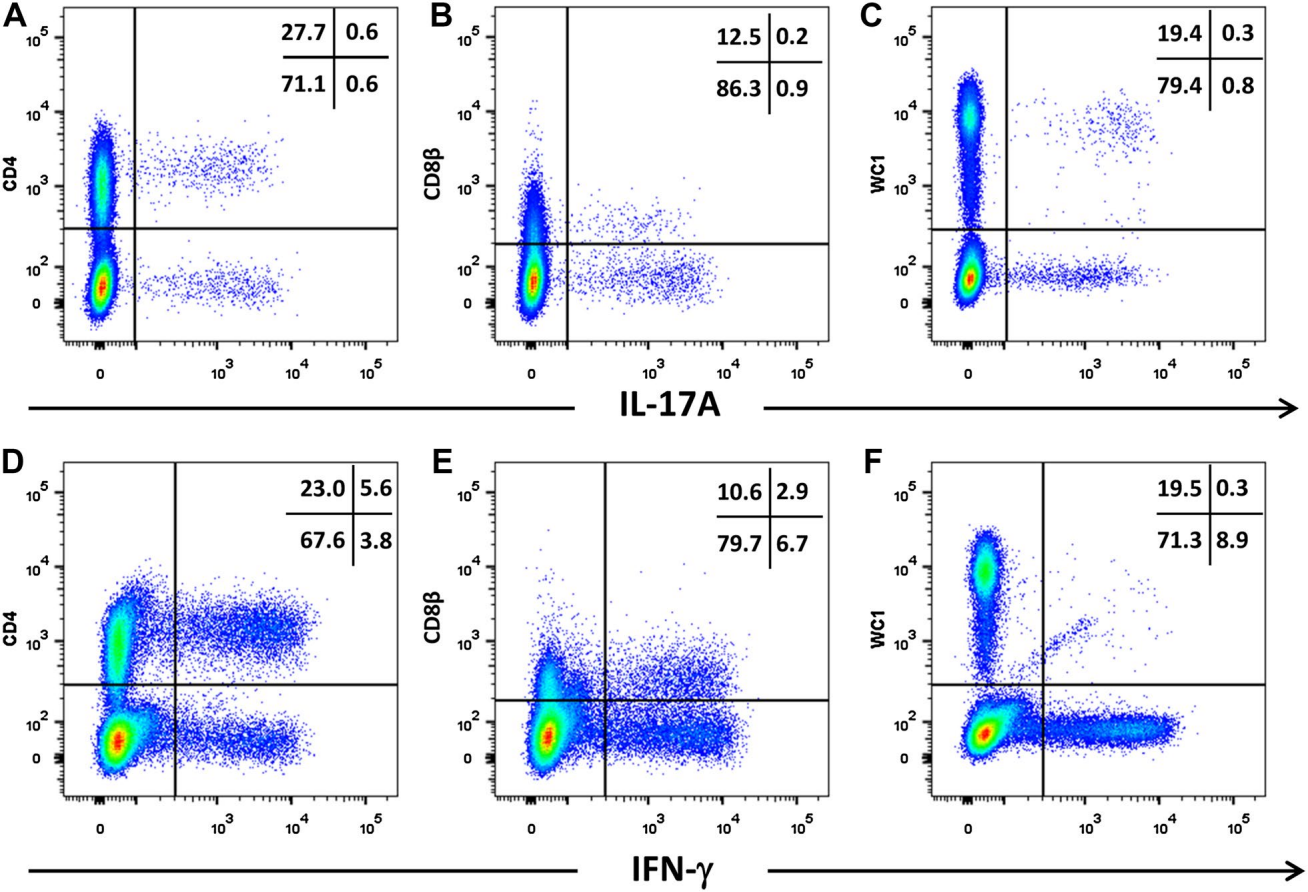
**Intracellular expression of IL-17A and IFN-γ by activated bovine T cell subsets.** PBMC from four cattle were stimulated with phorbol 12-myristate 13 acetate, ionomycin and brefeldin A in RPMI culture medium for 4 h. Cells were harvested and stained for viability and with mabs specific for cell-surface phenotypic markers and intracellular cytokines as described in Table 3 and “Expression of intracellular IL-17A and IFN-γ by bovine and ovine T cell subsets section”. Cells were stained for CD4 with mab CC8-PE at 1:20 dilution (**A**, **D**), for CD8β with mab CC58-PE at 1:20 dilution (**B**, **D**) and for WC-1 (γδ T cells) with mab CC15-PE at 1:200 (**C**, **E**). Intracellular cytokine staining for IL-17A was conducted using mab eBioDEC17-APC at a 1:20 dilution (**A**–**C**) and for IFN-γ using mab CC302-Alexafluor 647 at a 1:200 dilution (**D**–**F**). Data are shown for PBMC from one representative animal of four.

**Figure 6:**
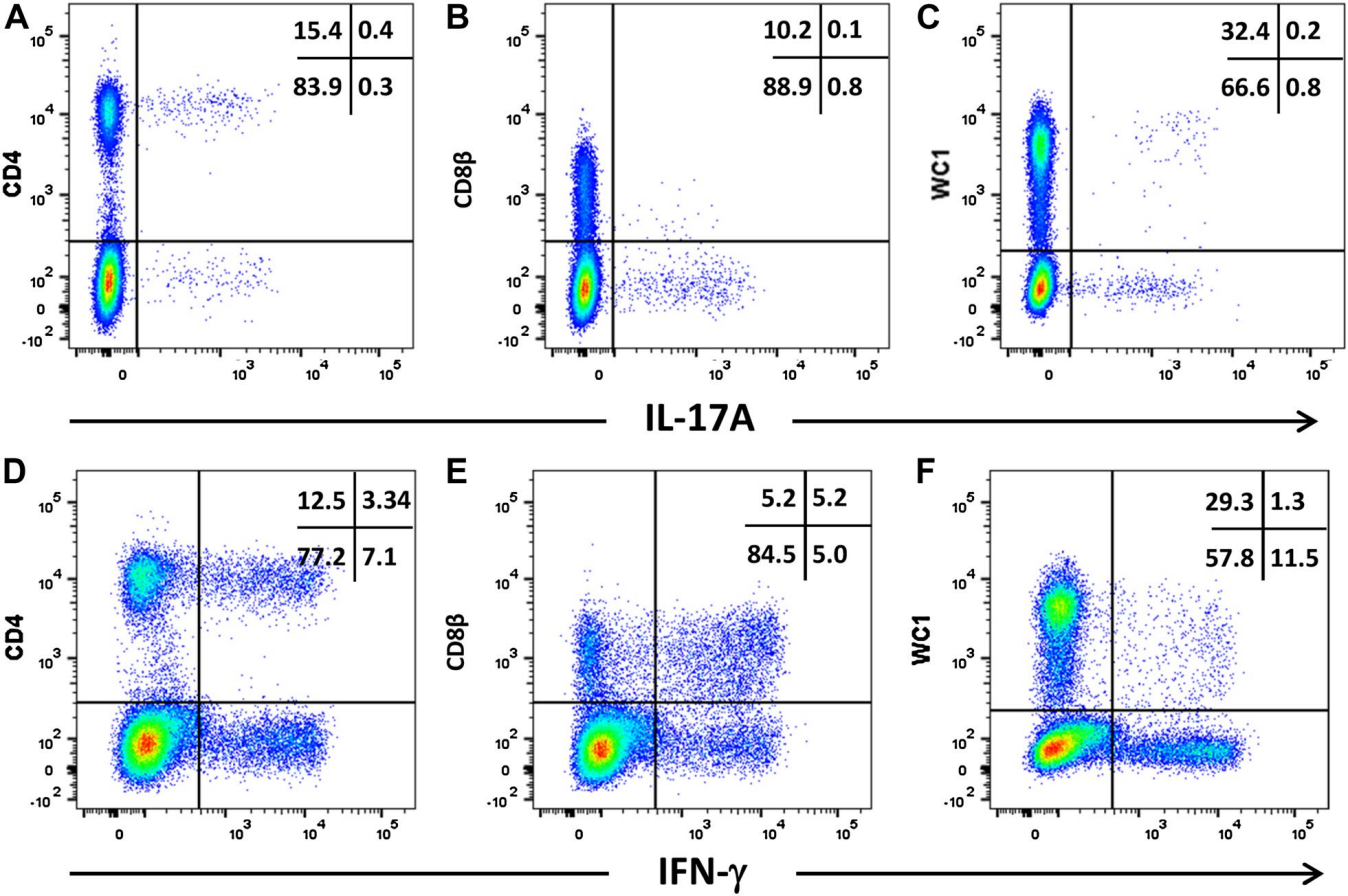
**Intracellular expression of IL-17A and IFN-γ by activated ovine T cell subsets.** PBMC from four sheep were stimulated with phorbol 12-myristate 13 acetate, ionomycin and brefeldin A in RPMI culture medium for 4 h. Cells were harvested and stained for viability and with mabs specific for cell-surface phenotypic markers and intracellular cytokines as described in Table 3 and “Expression of intracellular IL-17A and IFN-γ by bovine and ovine T cell subsets section”. Cells were then stained for CD4 with mab 44.38-PE at 1:20 dilution (**A**, **D**), CD8β with mab CC58-PE at 1:20 dilution (**B**, **D**) and WC-1 (γδ) with mab CC15-PE at 1:200 (**C**, **E**). Intracellular cytokine staining for IL-17A was conducted using mab eBio64DEC17-APC a 1:20 dilution (**A**–**C**) and for IFN-γ using mab CC302-alexafluor 647 at a 1:200 dilution (**D**–**F**). Data shown is for one representative animal out of four.

**Figure 7:**
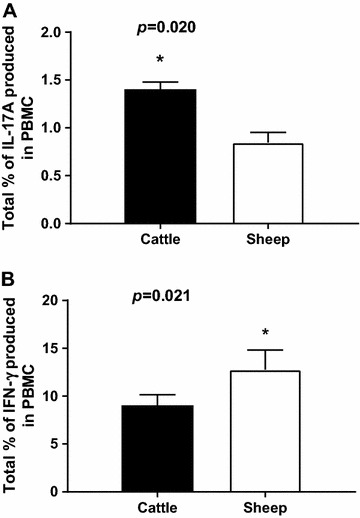
**Relative intracellular expression of IL-17A and IFN-γ by activated bovine and ovine PBMC.** The data sets described in “Expression of intracellular IL-17A and IFN-γ by bovine and ovine T cell subsets section” and presented in Figures 5 and 6 are summarised to compare overall intracellular expression of IL-17A (**A**) and IFN-γ (**B**) by PMA/ionomycin-stimulated bovine and ovine PBMC. Each bar represents the arithmetic mean of four cattle or four sheep and the error bars represent the standard error. The data for total percentage IFN-γ and IL-17A expression between species were assessed statistically using two-tailed Mann–Whitney tests allowing for ties.

PBMC from cattle and sheep were activated with PMA/ionomycin and the expression of intracellular cytokines by different T cells subsets was measured by double staining. Representative plots of the double staining of activated PBMC (one of four biological replicates) showed that CD4+ve, CD8β+ve (dim) and WC-1+ve cells from cattle whereas CD4+ve and WC-1+ve cells from sheep are capable of expressing intracellular IL-17A (Figures 5 and 6). Compared to the activated bovine PBMC, there are insufficient events to confirm the presence of CD8+ve(dim) cells expressing IL-17A in activated ovine PBMC. The proportions of cells expressing IL-17A are lower than those expressing IFN-γ in both cattle (Figure 5) and sheep (Figure 6), with the exception of the WC-1+ve cells in cattle which were proportionately greater for IL-17A. The bovine PBMC occupying the WC-1+ve IFN-γ+ve region in Figure 5F are mostly artefactual and non-specific (rectangular population crossing the region boundary for WC-1+ve region). However, under these stimulatory conditions, cattle PBMC proportionately expressed more IL-17A compared to sheep PBMC (Figure 7A; *p* = 0.020) whereas for IFN-γ the converse was true (Figure 7B; *p* = 0.021).

## Discussion

Since the discovery of two types of murine T cell clones with distinct cytokine expression [36], our knowledge of the cytokine profiles and function of the extended family of Th subsets has greatly expanded. In the process of gaining a deeper understanding of how the different CD4+ve Th subsets are induced to protect against different infections, it has become clear that they are no longer considered to be mutually-exclusive or irreversibly committed, but demonstrate a degree of plasticity and cross-regulation (reviewed by Zhu et al. [3]). Single-cell technologies are providing further insights into T cell biology and regulation of immune responses [37] which underpins novel strategic approaches to disease control and vaccine design.

Of the CD4+ve Th subsets described in mice, there appears to be a particular plasticity between Th-17 and Th-1 cells. In a model of diabetes purified Th-17 cells adoptively transferred into recipient mice have been shown to convert to Th-1-type cells secreting IFN-γ [38]. This close relationship of Th-1-type and Th-17-type responses and the cytokines that contribute to their differentiation, namely IL-12 and IL-23, that share the common p40 subunit, underlines the importance of being able to identify T cell cytokine profiles in target species. While the capability to study IFN-γ-related responses in farmed ruminants is advanced, there are relatively few reports describing the cellular expression and function of the IL-17 cytokine family.

Bougarn et al. [12] described the cloning of bovine IL-17A and IL-17F and the expression of biologically-active recombinant proteins in insect cells that could induce expression of a range of cytokines and chemokines (including CXCL8) in primary bovine mammary epithelial cells. We have taken the approach of comparing cattle and sheep by cloning and stably expressing bovine and ovine IL-17A in CHO cells. The rbovIL-17A and rovIL-17A were shown to be functionally active as they stimulated CXCL8 expression in fibroblasts and epithelial cells. The sequence identity of the mature bovine IL-17A protein with the ovine orthologue IL-17A is 100% which is reflected by their reciprocal species cross-reactivity (Figures 2B and C).

There have been several publications reporting the expression of mRNA encoding bovine IL-17A (and other IL-17 family members) as measured by RT-PCR [9, 39, 40]. There have also been publications reporting the quantification of IL-17A protein in cattle using the anti-bovIL-17A ELISA Vetset marketed by Kingfisher Biotech [10, 11, 39, 41, 42]. A principal objective of our study was to use the transfected CHO cells and their expressed recombinant proteins as defined standards to identify cross-reactive antibodies and to further refine methods to detect bovIL-17A and ovIL-17A. We have demonstrated for the first time the ability of the Kingfisher bovIL-17A Vetset to detect and quantify native ovIL-17A in the culture supernatants of mitogen-activated sheep PBMC (Figure 2D). The bovIL-17A Vetset ELISA antibodies have been used in an ELISpot to identify IL-17A-secreting CD4+ve and gamma-delta (γδ)T cells in cattle [43]. We have added to that capability by using these antibodies in an ELISpot to quantify the frequency of both bovIL-17A- and ovIL-17A-secreting cells in populations of activated PBMC (Figure 3). Our results show that PMA/ionomycin is a more potent stimulator of IL-17A in PBMC than ConA which is consistent with observations in human PBMC [44]. ConA induced a higher mean frequency of IL-17A-secreting cells from sheep PBMC than cattle, the reasons for which are unclear (Figure 3) but may include variability associated with age, breed and environment.

As described above, the ability to identify and quantify cytokine expression at the single-cell level by flow cytometry is invaluable for characterising cellular immune responses and informs on vaccine delivery strategies. The transfected CHO cells revealed that the bovIL-17A Vetset antibodies that were utilised successfully in ELISA and ELISpot were not optimal for intracellular IL-17A flow cytometric staining due to non-specific binding to untransfected cells (Figure 4A). We therefore focussed on screening a panel of seven anti-human or anti-mouse IL-17A mabs for ability to detect cattle and sheep IL-17A intracellularly. One of these mabs (eBio64DEC17) has been reported to detect intracellular IL-17A in sheep lymphocytes [45] and cattle, sheep and goat PBMC [46]. In these previous studies the cross-reactivity is implied as there was no validation against a specific control where IL-17A was known to be expressed. We have shown in a number of studies that stably transfected CHO cells constitutively express high levels of recombinant cytokines [30] and the transcription factor FoxP3 [31]. We have previously shown that brefeldin A increases intracellular accumulation of cytokine in the transfectants but does alter not the percentage positivity of the cells [47]. The intracellular cytokine staining of activated lymphocytes tends to be lower than the transfectants. Consequently, amplifying the signal even further in the CHO cells would not help select antibodies for native staining or give a better indication of their working dilutions for activated lymphocytes. In the present study, we found that eBio64DEC17 and another anti-human IL-17A mab (MT504) gave high deltaMFI values for bovIL-17A and ovIL-17A (Figures 4B and C), findings that were objectively confirmed using the PCA biplot analysis (Additional file 7). We demonstrate that deltaMFIs and to a lesser extent percentage of cells (in relation to appropriate control antibodies) can be used to objectively rank antibodies for their capacity to bind. MT504 clone is available in a tissue culture compatible format and was capable of neutralising rbovIL-17A and rovIL-17A function in vitro (Additional file 8). This mab adds to the panel of antibodies available to neutralise ruminant cytokine function which includes mabs 3C2 for ovine GMCSF [24], CC320 for bovine IL-10 [48] and CC326 for bovine IL-12 [49].

We compared intracellular staining for IL-17A using mab eBio64DEC17 with intracellular staining for IFN-γ in combination with cell-surface phenotyping to compare cytokine expression in activated cattle and sheep PBMC. Since the cytokines and the receptors involved in the production of IL-17A and IFN-γ share common components, it is important to know if distinctive T cell subsets expressing these cytokines exist in ruminants as they appear to in other mammalian species. We found that the major T cell subsets (CD4+ve and WC-1+ve) can express IL-17A (Figures 5 and 6), consistent with reports in humans [50] and mice [51, 52]. We found CD8+ve IL-17A for cattle PBMC but had insufficient cells to confirm their presence in PMA/ionomycin-stimulated ovine PBMC. Further evidence ideally in an antigen-driven setting is required to determine the existence of this cell population in sheep. There was a higher mean proportion of activated cattle PBMC expressing IL-17A than sheep PBMC, the converse being true for IFN-γ (Figure 7). The reason for this difference is unclear. The expression of bovine and ovine IL-17A by CD4+ve and WC-1+ve/γδT cell subsets is consistent with other studies using a combination of flow cytometry and ELISpot [43, 45]. Only 0.2% of total WC-1+ve/γδT cells from activated ovine PBMC expressed IL-17A in our study, whereas around 6% of total WC-1+ve/γδT cells were constitutively IL-17A+ve from skin-draining lymph. This difference is possibly reflective of the altered capacity for surveillance and mobilisation at the skin to cutaneous infection [45]. Our data show that activated bovine CD8+ve(dim) T cells also have the capacity to produce intracellular IL-17A, the first report of this phenomenon in ruminants, but consistent with reports of human CD8+ve T cells (reviewed by Srenathan et al. [51]).

IL-17A has an important role particularly at mucosal barriers such as the skin, lung and intestinal tract where its production can be crucial to the control or exacerbation of inflammation in humans or mice [53]. The new tools and assays developed in this study will enable greater investigation of mucosal compartments in ruminants to improve understanding of host response to infection in situ. The capability to measure both IL-17A and IFN-γ by intracellular staining will help determine if the Th-cell plasticity observed in Th-17 cells converting to a Th-1 IFN-γ-secreting phenotype in mice [38] can occur in ruminant species.

The validation and quality assurance of antibodies is currently a topic of great interest with several opinion articles discussing their impact on scientific advancement including inability to reproduce published data, the duplication of effort for testing reagents in specific assays and time lost due to use of unreliable reagents [54–56]. This is especially important in veterinary immunology as investment in immunological reagent development is relatively low [57].

In this study we have developed the capacity to measure native ovine IL-17A in liquid phase by ELISA and identify positive secreting cells by ELISpot using existing pabs from the commercial bovine ELISA. We have used molecular cloning techniques to generate stable mammalian CHO cells expressing recombinant IL-17A (constitutive positive and UTF negative controls) for screening a panel of commercial anti-IL-17A antibodies. Using PCA on six parameters we have represented the species cross-reactivity data identifying two suitable mabs for intracellular staining for sheep and cattle cells. We are able to identify CD4, CD8β and WC-1+ γδ T cells from activated bovine and ovine PBMC and block IL-17A activity. We took the approach of screening existing antibodies for detection of bovine and ovine IL-17A rather than embark on the production of new pabs or mabs against our recombinant proteins. This approach has the ethical advantage of avoiding immunisation schedules and therefore animal usage. There is always the possibility that higher affinity antibodies could be made against the homologous cytokine for each species by immunisation, but in balance we have shown that mabs produced against human cytokines can cross-react with ruminant cytokines. Confidence in the specific labelling is provided with the use of sufficiently robust controls. In summary, the tools we describe here for IL-17A will improve our ability to characterise inflammatory immune responses in ruminants and our understanding of host-pathogen interactions to inform on rational vaccine development.

## Additional files

**Additional file 1.** **Bovine, ovine and caprine IL-17 family sequences in publically accessible databases.** A list of the known IL-17 family orthologue sequences is shown where all sequences are full unless otherwise stated. The source of the sequence is stated either from mRNA/cDNA or predicted from genome annotation and the National Center for Biotechnology Information (NCBI, accessed 04/03/2017) accession numbers are as stated below: Cow (*Bos taurus*): IL-17-A EU682381.1; IL-17B NM_001192045.1; IL-17C XM_010826654.2; IL-17D transcript variant X1 XM_015465706.1; IL-17E/IL-25 XM_015464998.1 and IL-17F NM_001192082.1. Sheep (*Ovis aries*): IL-17A XM_004018887.3; IL-17B transcript variant X1 XM_012178770.2; IL-17C transcript variant X1 XM_012189660.2; IL-17D no accession record; IL-17E/IL-25 NM_001195219.1 and IL-17F XM_004018888.3. Goat (*Capra hircus*): IL-17A GU269912.1; IL-17B XM_005683151.3; IL-17C XM_005683151.3; IL-17D transcript variant X1 XM_018056543.1; IL-17E/IL-25 XM_018054603.1 and IL-17F XM_005696412.2.

**Additional file 2.** **Gating strategy used for the evaluation of commercial antibodies to bind intracellular recombinant bovine and ovine IL-17A in fixed cells.** Cells were acquired for flow cytometric analyses using the MacsQuant flow cytometer and analysed using the MacsQuantify Software. 20 000–50 000 events were collected and the following gating strategy was followed. Cells in the plot of Forward Scatter-Area (FSC-A) against the high dynamic range over time (HDR-T) are gated in P1 to exclude any non-specific artefacts (**A**). The P1/P2 gate represents Side Scatter-Area (SSC-A) plotted against FSC-A set to identify the main cell population and exclude debris (**B**). Single cells were gated (P1/P2/P3) using FSC-Height (H) vs FSC-A for doublet discrimination (**C**). Finally, the cells of interest were identified in the phycoerythrin or alexafluor 488 channel vs SSC-A (P1/P2/P3/P4) where regions were set using the isotype or equivalent control for each CHO cell line to establish threshold gates (**D**). Overlaying histogram plots of phycoerythrin or alexafluor 488 using (P1/P2/P3) gating strategy selecting for all cells in the region (equivalent to cells above and below region boundary in plot **D**) (**E**) were used to compare anti-IL-17A antibodies with appropriate isotype or equivalent controls presented in Figure 4. Gated percentage numbers above the region boundary (P1/P2/P3/P4) and median fluorescence region values (P1/P2/P3) were measured for each antibody in the relevant fluorochrome channel phycoerythrin or alexafluor 488. Delta median fluorescence intensity (deltaMFI) was calculated by deducting the median fluorescence region value for mab isotype control or pab control from the anti-IL-17A antibody value for the appropriate fluorochrome channel. The summarised data are presented in Additional file 3.

**Additional file 3.** **Summary of commercial IL-17A antibody evaluation for capacity to bind intracellular recombinant bovine and ovine IL-17A in fixed Chinese Hamster Ovary cells.** Control antibodies are listed in lower case, the anti-IL-17A antibodies are listed in upper case. Using the gating strategy described in brief in evaluation of commercial antibodies subsection (displayed in Additional file 2) the following data are shown: P1/P2/P3/P4 percentage of cells above the region boundary line of phycoerythrin or alexafluor 488 channels vs Side Scatter-Area (denoted #%); P1/P2/P3 median fluorescence region value (phycoerythrin or alexafluor 488 channels); and delta median fluorescence intensity (deltaMFI) calculated by taking the median fluorescence region values (phycoerythrin or alexafluor 488 channels) for the commercial IL-17A antibody and deducting the median fluorescence region value of the appropriate isotype or equivalent control antibody in the same fluorochrome channel) for the CHO UTF, bovIL-17A and ovIL-17A transfected, fixed CHO cells.

**Additional file 4.** **Gating strategy for the identification of activated bovine T cell subsets expressing intracellular IL-17A and IFN-γ.** Activated bovine PBMC were stained for viability, cell surface markers and intracellular cytokines according to the protocol outlined in “Expression of intracellular IL-17A and IFN-γ by bovine and ovine T cell subsets section” using antibodies listed in Table 3 and acquired using an LSRFortessa™ cell analyzer (Becton–Dickinson). Cells were gated to eliminate dead cells using the Vioblue Live/Dead^®^ Fixable Dead Cell Stain Kit, Side Scatter Height (SSC-H) vs Vioblue channel (A) and to include only single cells Forward Scatter Height (FSC-H) vs FSC-Area (FSC-A) (B). Gated single cells used for subsequent two-colour cell phenotyping and intracellular cytokine staining (C). Quadrant region boundaries were set based on isotype-matched directly conjugated antibody controls (FITC vs APC/Alexafluor 647 channels, D) and (Phycoerythrin vs APC/Alexafluor 647 channels, E) and fluorescence minus one (FMO) controls for each cell marker (WC-1, F; CD4, G; CD8β, H) and for each cytokine IL-17A, I-J and IFN-γ, K-L). Data are shown for one representative animal of four.

**Additional file 5.** **Gating strategy for the identification of activated ovine T cell subsets expressing intracellular IL-17A and IFN-γ.** Activated ovine PBMC were stained for viability, cell surface markers and intracellular cytokines according to the protocol outlined in “Expression of intracellular IL-17A and IFN-γ by bovine and ovine T cell subsets section” using antibodies listed in Table 3 and acquired using an LSRFortessa™ cell analyzer (Becton Dickenson). Cells were gated to eliminate dead cells using the Vioblue Live/Dead Stain^®^ Fixable Dead Cell Stain Kit, Side Scatter Height (SSC-H) vs Vioblue channel (A) and to include only single cells Forward Scatter Height (FSC-H) vs FSC-Area (FSC-A) (B). Gated single cells used for subsequent two-colour cell phenotyping and intracellular cytokine staining (C). Quadrant region boundaries were set based on isotype-matched directly conjugated antibody controls (FITC vs APC/Alexafluor 647 channels, D) and (Phycoerythrin vs APC/Alexafluor 647 channels, E) and fluorescence minus one (FMO) controls for each cell marker (WC-1, F; CD4, G; CD8β, H) and for each cytokine IL-17A, I-J and IFN-γ, K-L). Data are shown for one representative animal of four.

**Additional file 6.** **IL-17A sequence pair-wise identity matrix.** BovIL-17-A and ovIL-17A cDNAs encoding the mature proteins were aligned with the corresponding sequences from a variety of vertebrates including representative mammal, reptile and avian species and the protein pair-wise identity matrix was derived using Clustal 2.1.

**Additional file 7.** **Principal components analysis biplot of binding of commercial antibodies to transfected Chinese Hamster Ovary cells stably-expressing recombinant bovine or ovine IL-17A.** A principal component analysis (PCA) was conducted to investigate the structure of relationships between commercial monoclonal antibody clones to IL-17A and the six metrics used to assess antibody staining. These metrics were the binding to transfected CHO cells stably expressing rbovIL-17A, rovIL-17A or the untransfected CHO negative control (UTF) cells [numerical percentage of (%) cells] in the upper positive region (% CHO UTF, % bovIL-17A and % ovIL-17A) and delta median fluorescence intensity (deltaMFI) values for the same three CHO cell lines. Data used in the PCA is taken from Additional File 6. PCA reduced the dimension of the data set by means of optimal linear combinations (principal components, PCs) of the six metrics aimed to retain as much of the original data variability as possible. The results were displayed using a correlation biplot based on the two first PCs (those accounting for the highest percentage of the total variability) to facilitate discussion and ranking of the commercial antibodies.

**Additional file 8.** **Neutralisation of recombinant bovine and ovine IL-17A activity on ovine cells by commercial mab.** Ovine ST-6 cells were set up as described in “Bulk recombinant cytokine production and functional determination of recombinant bovine and ovine IL-17A section” but using 96 well flat bottom plates. The following treatments were pre-incubated at 37 °C for 2 h in a water bath: IMDM culture medium only (unstimulated cells), rovIL-17A (50 ng/mL), rovIL-17A (50 ng/mL) + MT504 monoclonal antibody (mab, 1 μg/mL), rbovIL-17A (50 ng/mL) and rbovIL-17A (50 ng/mL) + MT504 mab (1 μg/mL). The treatments were then added to the ovine cells for 24 h and harvested and assayed as previously described. The X-axis displays the neutralisation bioassay treatments and the Y-axis shows levels of CXCL8 in pg/mL. Data are the arithmetic mean of three technical replicate samples from one representative experiment of two. The percentage neutralisation values displayed on the graph have been calculated by firstly deducting the unstimulated (IMDM culture medium control) value from all other treatment values. The value for rbovIL-17A neutralisation with MT504 mab was calculated by: 100 minus [(rbovIL-17A/MT504 mab minus background value) divided by (rbovIL-17A minus background value) multiplied by 100]. Percentage neutralisation for rovIL-17A was calculated by substituting rbovIL-17A for rovIL-17A values into equation above.
