# Supplementary material for: Enhancing the toolbox to study IL-17A in cattle and sheep
Source: Vet Res. 2017 Apr 8;48:20. doi: 10.1186/s13567-017-0426-5 (PMC5385008; doi:10.1186/s13567-017-0426-5)
Supplement: Supplementary file 1 — Additional file 1. Bovine, ovine and caprine IL-17 family sequences in publically accessible databases. A list of the known IL-17 family orthologue sequences is shown where all sequences are full unless otherwise stated. The source of the sequence is stated either from mRNA/cDNA or predicted from genome annotation and the National Center for Biotechnology Information (NCBI, accessed 04/03/2017) accession numbers are as stated below: Cow (Bos taurus): IL-17-A EU682381.1; IL-17B NM_001192045.1; IL-17C XM_010826654.2; IL-17D transcript variant X1 XM_015465706.1; IL-17E/IL-25 XM_015464998.1 and IL-17F NM_001192082.1. Sheep (Ovis aries): IL-17A XM_004018887.3; IL-17B transcript variant X1 XM_012178770.2; IL-17C transcript variant X1 XM_012189660.2; IL-17D no accession record; IL-17E/IL-25 NM_001195219.1 and IL-17F XM_004018888.3. Goat (Capra hircus): IL-17A GU269912.1; IL-17B XM_005683151.3; IL-17C XM_005683151.3; IL-17D transcript variant X1 XM_018056543.1; IL-17E/IL-25 XM_018054603.1 and IL-17F XM_005696412.2. [file 13567_2017_426_MOESM1_ESM.pdf]

	Bovine sequence source		Ovine sequence source		Caprine sequence source	
IL-17 family orthologue	mRNA or cDNA	Predicted from genome annotation	mRNA or cDNA	Predicted from genome annotation	mRNA or cDNA	Predicted from genome annotation
IL-17A	✓			✓	✓	
IL-17B		✓		✓		✓
IL-17C		✓ partial		✓		✓
IL-17D		✓				✓
IL-17E/ IL-25		✓	✓			✓
IL-17F		✓		✓		✓
