# Supplementary figures and images for: Enhancing the toolbox to study IL-17A in cattle and sheep
Source: Vet Res. 2017 Apr 8;48:20. doi: 10.1186/s13567-017-0426-5 (PMC5385008; doi:10.1186/s13567-017-0426-5)

A

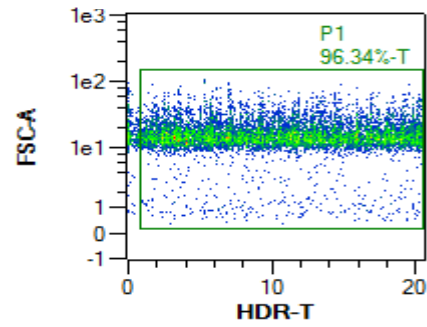

B

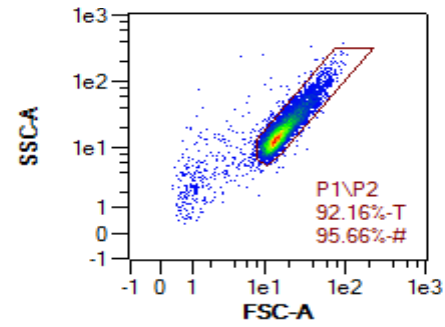

C

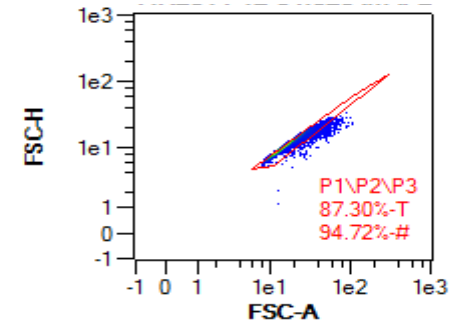

D

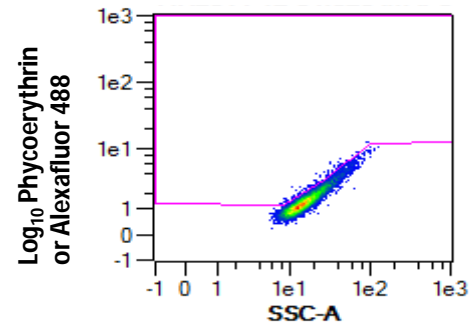

E

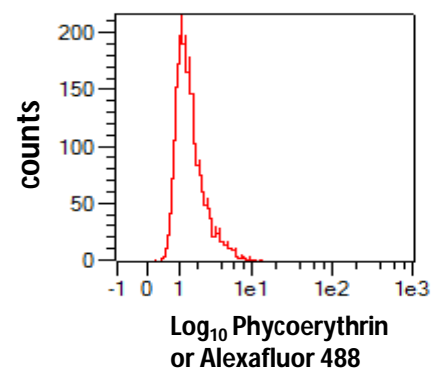

Supplement: Supplementary file 2 — Additional file 2. Gating strategy used for the evaluation of commercial antibodies to bind intracellular recombinant bovine and ovine IL-17A in fixed cells. Cells were acquired for flow cytometric analyses using the MacsQuant flow cytometer and analysed using the MacsQuantify Software. 20 000–50 000 events were collected and the following gating strategy was followed. Cells in the plot of Forward Scatter-Area (FSC-A) against the high dynamic range over time (HDR-T) are gated in P1 to exclude any non-specific artefacts (A). The P1/P2 gate represents Side Scatter-Area (SSC-A) plotted against FSC-A set to identify the main cell population and exclude debris (B). Single cells were gated (P1/P2/P3) using FSC-Height (H) vs FSC-A for doublet discrimination (C). Finally, the cells of interest were identified in the phycoerythrin or alexafluor 488 channel vs SSC-A (P1/P2/P3/P4) where regions were set using the isotype or equivalent control for each CHO cell line to establish threshold gates (D). Overlaying histogram plots of phycoerythrin or alexafluor 488 using (P1/P2/P3) gating strategy selecting for all cells in the region (equivalent to cells above and below region boundary in plot D) (E) were used to compare anti-IL-17A antibodies with appropriate isotype or equivalent controls presented in Figure 4. Gated percentage numbers above the region boundary (P1/P2/P3/P4) and median fluorescence region values (P1/P2/P3) were measured for each antibody in the relevant fluorochrome channel phycoerythrin or alexafluor 488. Delta median fluorescence intensity (deltaMFI) was calculated by deducting the median fluorescence region value for mab isotype control or pab control from the anti-IL-17A antibody value for the appropriate fluorochrome channel. The summarised data are presented in Additional file 3. [file 13567_2017_426_MOESM2_ESM.pdf]

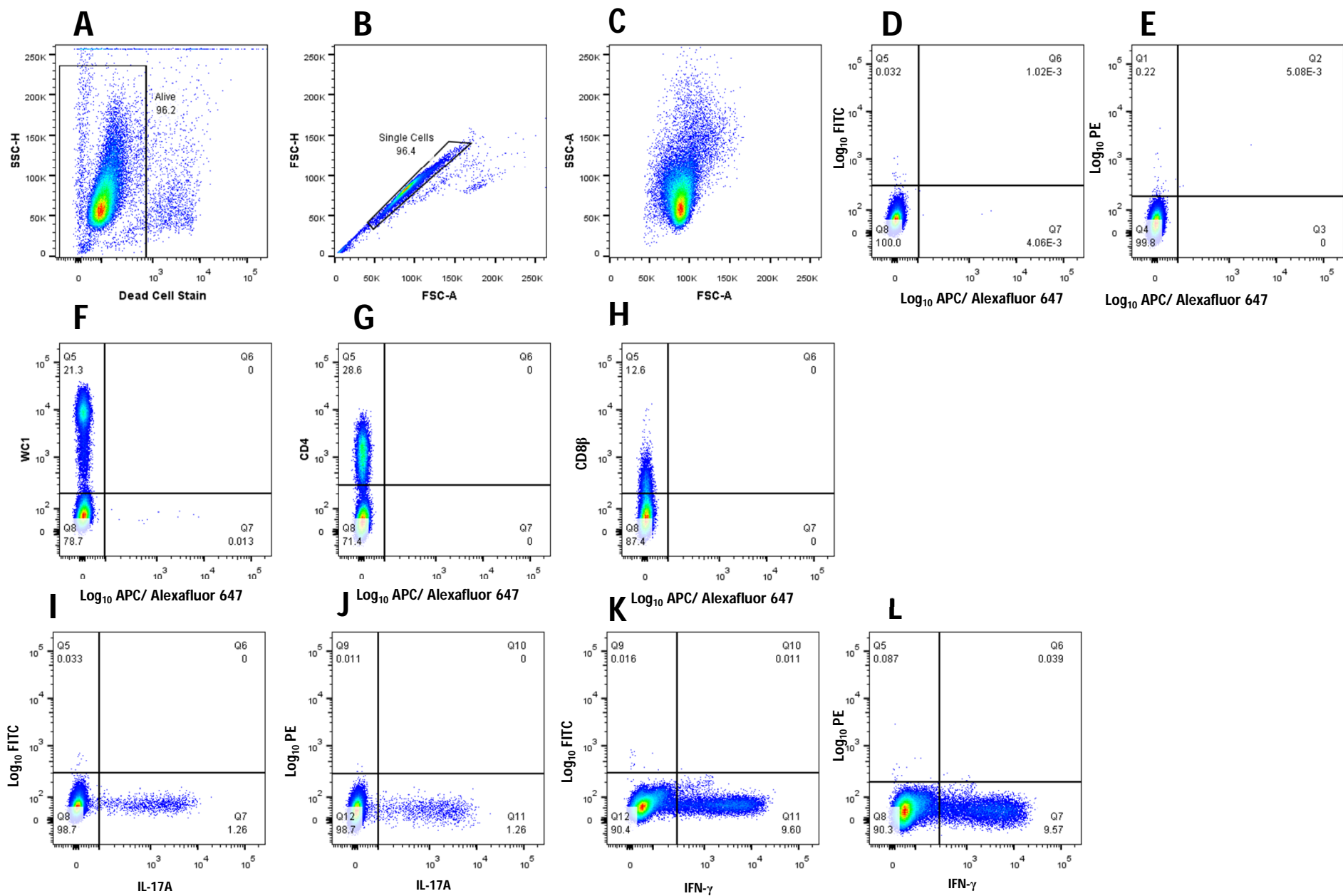

Supplement: Supplementary file 4 — Additional file 4. Gating strategy for the identification of activated bovine T cell subsets expressing intracellular IL-17A and IFN-γ. Activated bovine PBMC were stained for viability, cell surface markers and intracellular cytokines according to the protocol outlined in “Expression of intracellular IL-17A and IFN-γ by bovine and ovine T cell subsets section” using antibodies listed in Table 3 and acquired using an LSRFortessa™ cell analyzer (Becton–Dickinson). Cells were gated to eliminate dead cells using the Vioblue Live/Dead® Fixable Dead Cell Stain Kit, Side Scatter Height (SSC-H) vs Vioblue channel (A) and to include only single cells Forward Scatter Height (FSC-H) vs FSC-Area (FSC-A) (B). Gated single cells used for subsequent two-colour cell phenotyping and intracellular cytokine staining (C). Quadrant region boundaries were set based on isotype-matched directly conjugated antibody controls (FITC vs APC/Alexafluor 647 channels, D) and (Phycoerythrin vs APC/Alexafluor 647 channels, E) and fluorescence minus one (FMO) controls for each cell marker (WC-1, F; CD4, G; CD8β, H) and for each cytokine IL-17A, I-J and IFN-γ, K-L). Data are shown for one representative animal of four. [file 13567_2017_426_MOESM4_ESM.pdf]

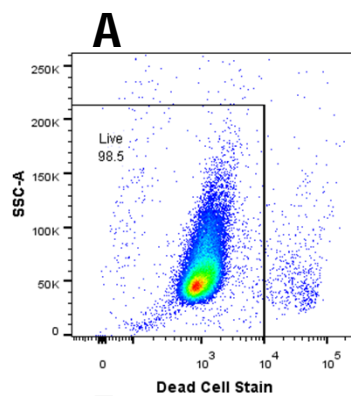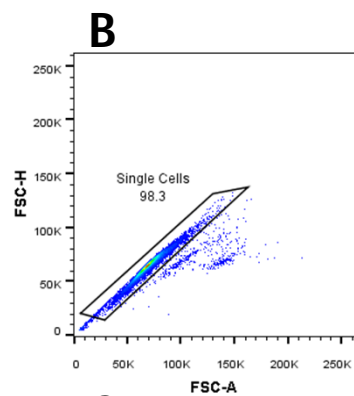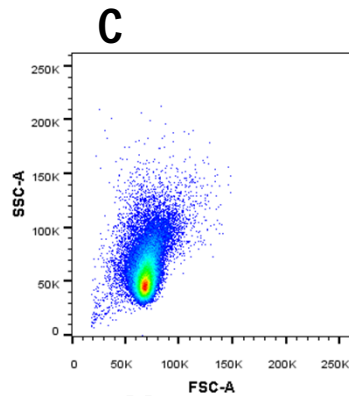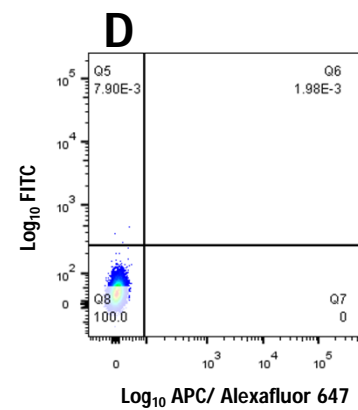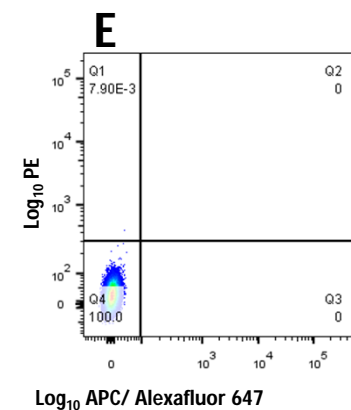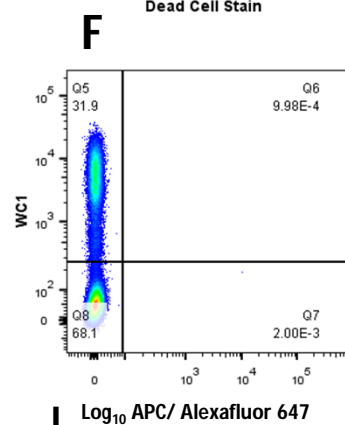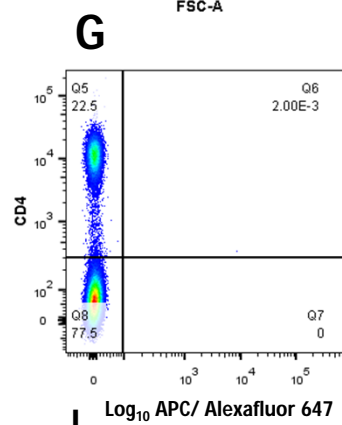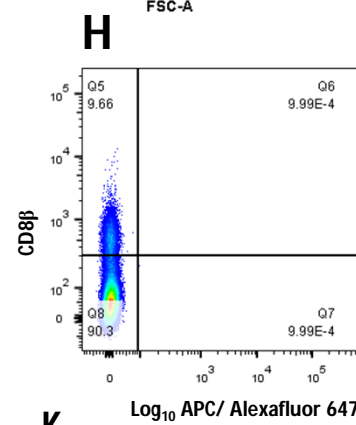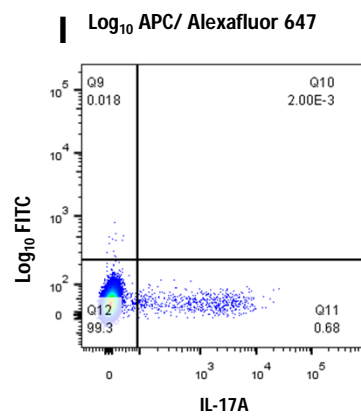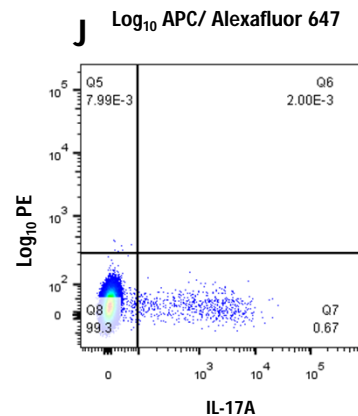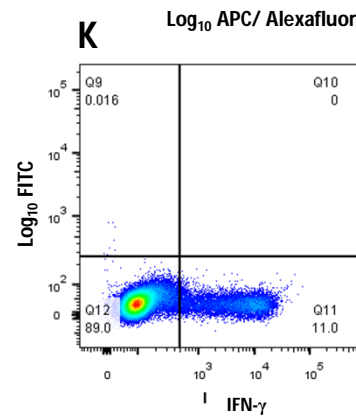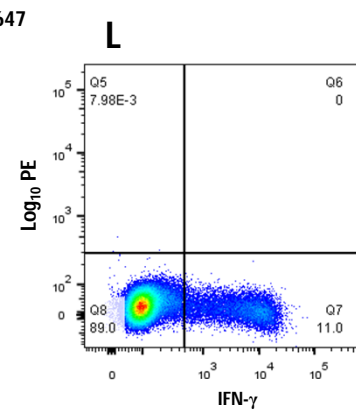

Supplement: Supplementary file 5 — Additional file 5. Gating strategy for the identification of activated ovine T cell subsets expressing intracellular IL-17A and IFN-γ. Activated ovine PBMC were stained for viability, cell surface markers and intracellular cytokines according to the protocol outlined in “Expression of intracellular IL-17A and IFN-γ by bovine and ovine T cell subsets section” using antibodies listed in Table 3 and acquired using an LSRFortessa™ cell analyzer (Becton Dickenson). Cells were gated to eliminate dead cells using the Vioblue Live/Dead Stain® Fixable Dead Cell Stain Kit, Side Scatter Height (SSC-H) vs Vioblue channel (A) and to include only single cells Forward Scatter Height (FSC-H) vs FSC-Area (FSC-A) (B). Gated single cells used for subsequent two-colour cell phenotyping and intracellular cytokine staining (C). Quadrant region boundaries were set based on isotype-matched directly conjugated antibody controls (FITC vs APC/Alexafluor 647 channels, D) and (Phycoerythrin vs APC/Alexafluor 647 channels, E) and fluorescence minus one (FMO) controls for each cell marker (WC-1, F; CD4, G; CD8β, H) and for each cytokine IL-17A, I-J and IFN-γ, K-L). Data are shown for one representative animal of four. [file 13567_2017_426_MOESM5_ESM.pdf]

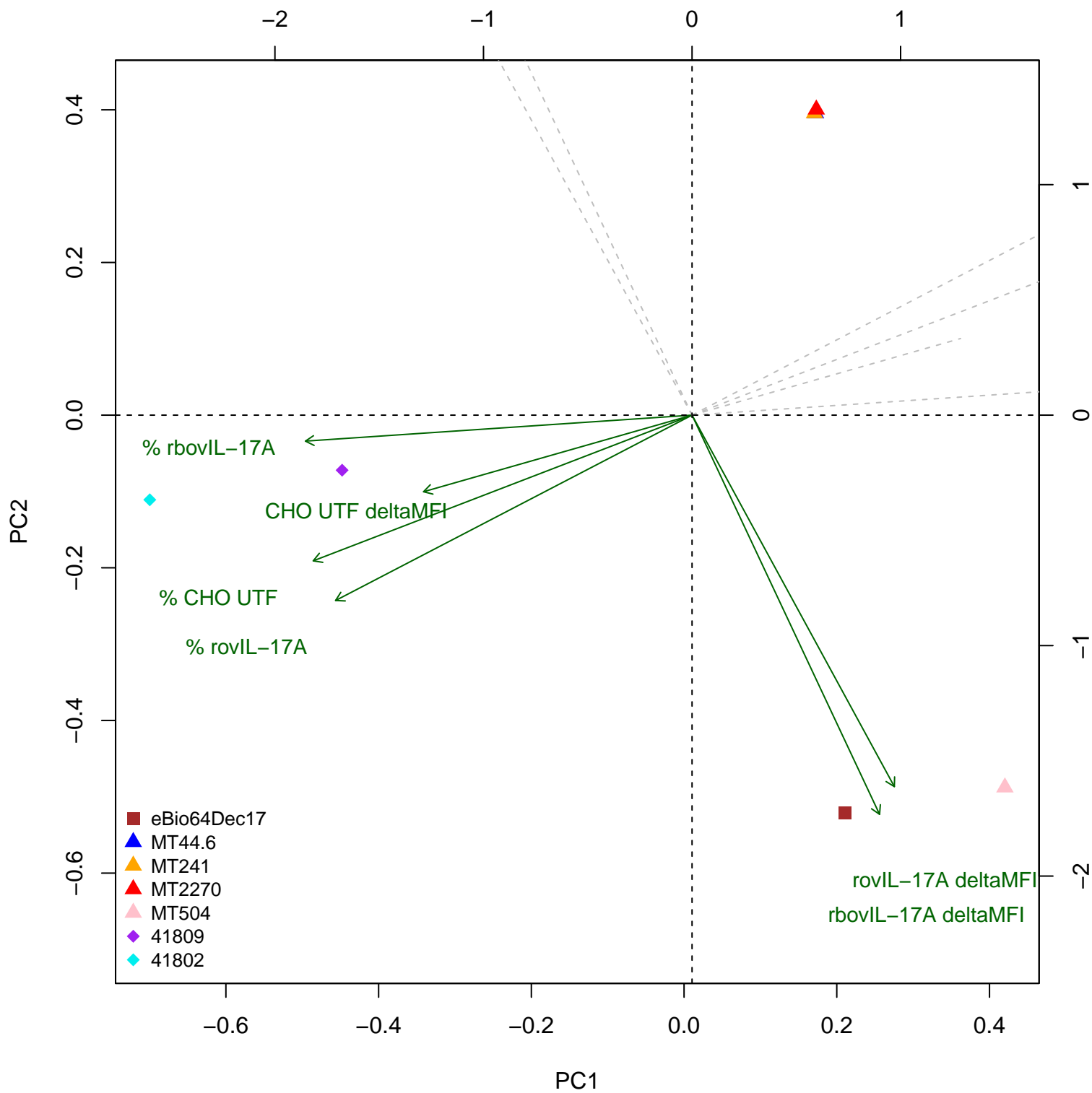

Supplement: Supplementary file 7 — Additional file 7. Principal components analysis biplot of binding of commercial antibodies to transfected Chinese Hamster Ovary cells stably-expressing recombinant bovine or ovine IL-17A. A principal component analysis (PCA) was conducted to investigate the structure of relationships between commercial monoclonal antibody clones to IL-17A and the six metrics used to assess antibody staining. These metrics were the binding to transfected CHO cells stably expressing rbovIL-17A, rovIL-17A or the untransfected CHO negative control (UTF) cells [numerical percentage of (%) cells] in the upper positive region (% CHO UTF, % bovIL-17A and % ovIL-17A) and delta median fluorescence intensity (deltaMFI) values for the same three CHO cell lines. Data used in the PCA is taken from Additional File 6. PCA reduced the dimension of the data set by means of optimal linear combinations (principal components, PCs) of the six metrics aimed to retain as much of the original data variability as possible. The results were displayed using a correlation biplot based on the two first PCs (those accounting for the highest percentage of the total variability) to facilitate discussion and ranking of the commercial antibodies. [file 13567_2017_426_MOESM7_ESM.pdf]

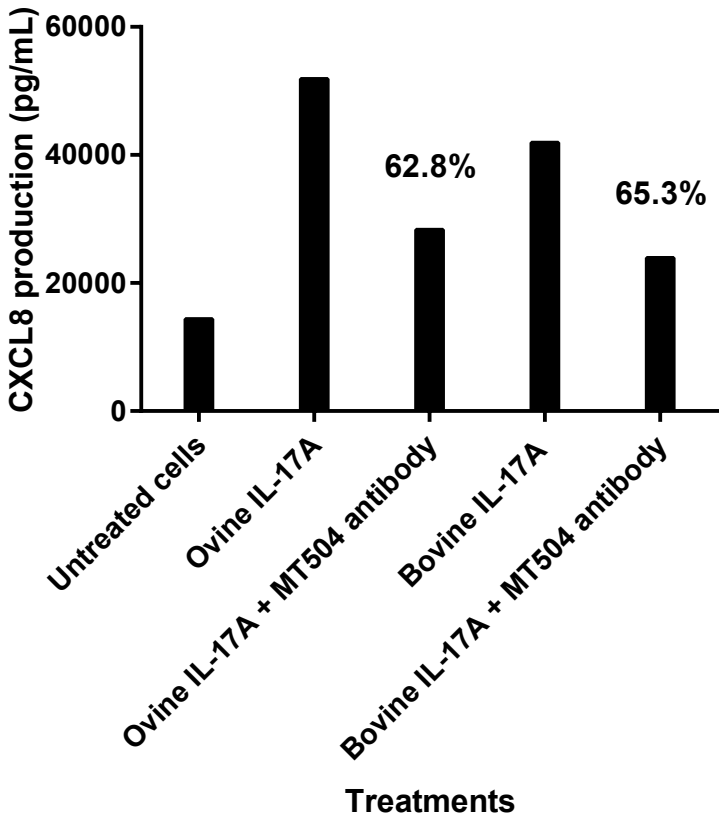

Supplement: Supplementary file 8 — Additional file 8. Neutralisation of recombinant bovine and ovine IL-17A activity on ovine cells by commercial mab. Ovine ST-6 cells were set up as described in “Bulk recombinant cytokine production and functional determination of recombinant bovine and ovine IL-17A section” but using 96 well flat bottom plates. The following treatments were pre-incubated at 37 °C for 2 h in a water bath: IMDM culture medium only (unstimulated cells), rovIL-17A (50 ng/mL), rovIL-17A (50 ng/mL) + MT504 monoclonal antibody (mab, 1 μg/mL), rbovIL-17A (50 ng/mL) and rbovIL-17A (50 ng/mL) + MT504 mab (1 μg/mL). The treatments were then added to the ovine cells for 24 h and harvested and assayed as previously described. The X-axis displays the neutralisation bioassay treatments and the Y-axis shows levels of CXCL8 in pg/mL. Data are the arithmetic mean of three technical replicate samples from one representative experiment of two. The percentage neutralisation values displayed on the graph have been calculated by firstly deducting the unstimulated (IMDM culture medium control) value from all other treatment values. The value for rbovIL-17A neutralisation with MT504 mab was calculated by: 100 minus [(rbovIL-17A/MT504 mab minus background value) divided by (rbovIL-17A minus background value) multiplied by 100]. Percentage neutralisation for rovIL-17A was calculated by substituting rbovIL-17A for rovIL-17A values into equation above. [file 13567_2017_426_MOESM8_ESM.pdf]
