# Supplementary material for: Enhancing the toolbox to study IL-17A in cattle and sheep
Source: Vet Res. 2017 Apr 8;48:20. doi: 10.1186/s13567-017-0426-5 (PMC5385008; doi:10.1186/s13567-017-0426-5)
Supplement: Supplementary file 3 — Additional file 3. Summary of commercial IL-17A antibody evaluation for capacity to bind intracellular recombinant bovine and ovine IL-17A in fixed Chinese Hamster Ovary cells. Control antibodies are listed in lower case, the anti-IL-17A antibodies are listed in upper case. Using the gating strategy described in brief in evaluation of commercial antibodies subsection (displayed in Additional file 2) the following data are shown: P1/P2/P3/P4 percentage of cells above the region boundary line of phycoerythrin or alexafluor 488 channels vs Side Scatter-Area (denoted #%); P1/P2/P3 median fluorescence region value (phycoerythrin or alexafluor 488 channels); and delta median fluorescence intensity (deltaMFI) calculated by taking the median fluorescence region values (phycoerythrin or alexafluor 488 channels) for the commercial IL-17A antibody and deducting the median fluorescence region value of the appropriate isotype or equivalent control antibody in the same fluorochrome channel) for the CHO UTF, bovIL-17A and ovIL-17A transfected, fixed CHO cells. [file 13567_2017_426_MOESM3_ESM.pdf]

Primary antibody code	Antibody Clone isotype for mAbs in brackets	Supplier / product code	Antibody conjugation (where present)	Fluorochrome for detection antibody	CHO UTF			Bovine IL-17A			Ovine IL-17A		
					# %	Median Fluorescence Region value	Delta Median Fluorescence Intensity (deltaMFI)	# %	Median Fluorescence Region value	deltaMFI	# %	Median Fluorescence Region value	deltaMFI
(a)	N/A (pAb)	Roslin Institute/ N/A	N/A	AlexaFluor 488	0.67	1.23	0	0.86	3.72	0	0.66	3.52	0
(A)	N/A (pAb)	Kingfisher Biotech/ PB0274B-100	N/A	AlexaFluor 488	96.15	3.46	2.23	99.49	17.36	13.64	99.86	46.62	43.1
(b)	VPM21 (IgG1)	Moredun/ N/A	N/A	R-Phycoerythrin	1.17	0.4	0	0.39	1.55	0	0.92	1.34	0
(B)	eBio64Dec17 (IgG1)	e-Bioscience/ 12-7179	R-PE	R-Phycoerythrin	12.54	0.51	0.11	38.74	2.58	1.03	92.93	7.41	6.07
(c)	VPM21 (IgG1)	Moredun/ N/A	N/A	R-Phycoerythrin	0.38	0.44	0	0.46	0.99	0	0.31	1.48	0
(C.1)	MT44.6 (IgG1)	Mabtech/ 3520-3-250	N/A	R-Phycoerythrin	0.57	0.42	0	0.51	0.99	0	0.46	1.56	0.08
(C.2)	MT241 (IgG1)	Mabtech/ 3520M-3-250	N/A	R-Phycoerythrin	0.38	0.43	0	0.53	0.99	0	0.36	1.55	0.07
(C.3)	MT2270	Mabtech/ 3521-14-250	N/A	R-Phycoerythrin	0.29	0.4	0	0.36	0.99	0	0.32	1.5	0.02
(C.4)	MT504 (IgG1)	Mabtech/ 3520-5N-500	Biotin	R-Phycoerythrin	0.76	0.49	0.05	61.74	2.26	1.27	95.96	8.2	6.72
(d)	VPM21 (IgG1)/ VPM22 (IgG2b)	Moredun/ N/A	N/A	R-Phycoerythrin	2.55	0.61	0	0.79	0.58	0	1.37	1.17	0
(D.1)	41809 (IgG2b)	R & D Systems/ IC317P	R-PE	R-Phycoerythrin	0.06	0.37	0	17.73	0.72	0.14	78.58	2.33	1.12
(D.2)	41802 (IgG1)	R & D Systems/ IC3171P	R-PE	R-Phycoerythrin	94.14	2.45	1.84	4.07	0.69	0.11	5.35	1.37	0.2

Control antibodies are listed in lower case, the anti-IL-17A antibodies are listed in upper case. Using the gating strategy described in brief in evaluation of commercial antibodies subsection (displayed in Additional file 3) the following data are shown: P1/P2/P3/P4 percentage of cells above the region boundary line of phycoerythrin or alexafluor 488 channels vs Side Scatter-Area (denoted #%); P1/P2/P3 median fluorescence region value (phycoerythrin or alexafluor 488 channels); and delta median fluorescence intensity (deltaMFI) calculated by taking the median fluorescence region values (phycoerythrin or alexafluor 488 channels) for the commercial IL-17A antibody and deducting the median fluorescence region value of the appropriate isotype or equivalent control antibody in the same fluorochrome channel) for the CHO UTF, bovIL-17A and ovIL-17A transfected, fixed CHO cells.
