# Supplementary material for: Enhancing the toolbox to study IL-17A in cattle and sheep
Source: Vet Res. 2017 Apr 8;48:20. doi: 10.1186/s13567-017-0426-5 (PMC5385008; doi:10.1186/s13567-017-0426-5)
Supplement: Supplementary file 6 — Additional file 6. IL-17A sequence pair-wise identity matrix. BovIL-17-A and ovIL-17A cDNAs encoding the mature proteins were aligned with the corresponding sequences from a variety of vertebrates including representative mammal, reptile and avian species and the protein pair-wise identity matrix was derived using Clustal 2.1. [file 13567_2017_426_MOESM6_ESM.pdf]

	Koala	Chinese hamster	Common Brown Rat	House Mouse	Naked mole rat	Domestic guinea pig	Pig	Wild bactrian camel	Goat	Cow	Sheep	Chinese tree shrew	Brandts bat	David Myotis Vesper bat	Human	Black flying fox	Horse	Pygmy rabbit	European hare	European rabbit	Brush rabbit	Lesser panda	Dog	American alligator	Mallard wild duck	Chicken
Koala	100																									
Chinese hamster	54.84	100																								
Common Brown Rat	54.03	89.52	100																							
House Mouse	52.42	87.9	91.13	100																						
Naked mole rat	58.06	72.58	66.13	67.74	100																					
Domestic guinea pig	57.26	70.97	66.94	66.94	89.52	100																				
Pig	58.06	72.58	68.55	67.74	77.42	71.77	100																			
Wild bactrian camel	57.26	73.39	69.35	71.77	76.61	74.19	83.06	100																		
Goat	60.48	73.39	70.16	70.97	76.61	70.97	84.68	88.71	100																	
Cow	61.29	74.19	70.97	71.77	77.42	71.77	85.48	89.52	99.19	100																
Sheep	61.29	74.19	70.97	71.77	77.42	71.77	85.48	89.52	99.19	100	100															
Chinese tree shrew	58.06	68.55	64.52	63.71	72.58	71.77	75.81	78.23	77.42	78.23	78.23	100														
Brandts bat	57.26	70.97	67.74	67.74	73.39	70.16	79.03	77.42	78.23	78.23	78.23	79.84	100													
David Myotis Vesper bat	58.06	70.97	67.74	67.74	71.77	69.35	75.81	74.19	74.19	75	75	76.61	91.13	100												
Human	60.48	67.74	64.52	64.52	74.19	70.16	76.61	78.23	75.81	76.61	76.61	78.23	79.84	77.42	100											
Black flying fox	57.26	75	70.16	70.16	72.58	70.16	79.03	79.84	77.42	78.23	78.23	77.42	83.06	81.45	80.65	100										
Horse	57.26	77.42	71.77	71.77	76.61	73.39	75.81	80.65	81.45	82.26	82.26	79.84	79.84	79.84	78.23	83.06	100									
Pygmy rabbit	61.29	73.39	71.77	69.35	72.58	71.77	75.81	81.45	82.26	83.06	83.06	79.03	77.42	76.61	79.84	83.87	85.48	100								
European hare	60.48	71.77	70.16	67.74	72.58	72.58	75	79.84	81.45	82.26	82.26	80.65	78.23	77.42	80.65	79.84	82.26	95.16	100							
European rabbit	59.68	73.39	70.97	69.35	75	74.19	76.61	81.45	81.45	82.26	82.26	81.45	79.84	78.23	82.26	82.26	84.68	95.97	97.58	100						
Brush rabbit	60.48	73.39	71.77	69.35	75	74.19	76.61	82.26	83.06	83.87	83.87	81.45	79.03	78.23	81.45	81.45	83.87	96.77	96.77	97.58	100					
Lesser panda	54.84	72.58	68.55	69.35	74.19	70.16	78.23	82.26	81.45	82.26	82.26	81.45	79.03	76.61	78.23	82.26	84.68	85.48	83.87	56.29	85.48	100				
Dog	54.84	73.39	69.35	69.35	75	70.97	78.23	82.26	82.26	83.06	83.06	81.45	81.45	79.03	81.45	85.48	86.29	87.9	86.29	88.71	87.9	93.55	100			
American alligator	44.35	44.35	43.55	43.55	42.74	43.55	44.35	45.16	41.94	42.74	42.74	47.58	46.77	48.39	50	47.58	49.19	46.77	47.58	48.39	47.58	46.77	47.58	100		
Mallard wild duck	45.16	45.97	41.94	41.13	47.58	50.81	49.19	50.81	48.39	49.19	49.19	49.19	50	51.61	47.58	47.58	45.16	47.58	49.19	49.19	49.19	45.16	47.58	57.26	100	
Chicken	46.77	45.16	42.74	41.94	46.77	49.19	50	50.81	47.58	48.39	48.39	48.39	49.19	51.61	47.58	46.77	44.35	46.77	48.39	48.39	48.39	44.35	46.77	55.65	87.9	100

BovIL-17-A and ovIL-17A cDNAs encoding the mature proteins were aligned with the corresponding sequences from a variety of vertebrates including representative mammal, reptile and avian species and the protein pair-wise identity matrix was derived using Clustal 2.1.
